# Optimizing a Multi-Component Intranasal *Entamoeba Histolytica* Vaccine Formulation Using a Design of Experiments Strategy

**DOI:** 10.3389/fimmu.2021.683157

**Published:** 2021-06-25

**Authors:** Mayuresh M. Abhyankar, Mark T. Orr, Robert Kinsey, Sandra Sivananthan, Andrew J. Nafziger, David N. Oakland, Mary K. Young, Laura Farr, Md Jashim Uddin, Jhansi L. Leslie, Stacey L. Burgess, Hong Liang, Ines De Lima, Elise Larson, Jeffrey A. Guderian, Susan Lin, Aaron Kahn, Prakash Ghosh, Sierra Reed, Mark A. Tomai, Karl Pedersen, William A. Petri, Christopher B. Fox

**Affiliations:** ^1^ Division of Infectious Diseases and International Health, Department of Medicine, University of Virginia Health System, Charlottesville, VA, United States; ^2^ Infectious Disease Research Institute (IDRI), Seattle, WA, United States; ^3^ Department of Global Health, University of Washington, Seattle, WA, United States; ^4^ 3M Corporate Research Materials Laboratory, 3M Center, St Paul, MN, United States; ^5^ TECHLAB, Inc., Blacksburg, VA, United States

**Keywords:** *Entamoeba histolytica*, vaccine adjuvant, TLR ligand, intranasal, liposome, formulation, design of experiments

## Abstract

Amebiasis is a neglected tropical disease caused by *Entamoeba histolytic*a. Although the disease burden varies geographically, amebiasis is estimated to account for some 55,000 deaths and millions of infections globally per year. Children and travelers are among the groups with the greatest risk of infection. There are currently no licensed vaccines for prevention of amebiasis, although key immune correlates for protection have been proposed from observational studies in humans. We previously described the development of a liposomal adjuvant formulation containing two synthetic TLR ligands (GLA and 3M-052) that enhanced antigen-specific fecal IgA, serum IgG2a, a mixed IFNγ and IL-17A cytokine profile from splenocytes, and protective efficacy following intranasal administration with the LecA antigen. By applying a statistical design of experiments (DOE) and desirability function approach, we now describe the optimization of the dose of each vaccine formulation component (LecA, GLA, 3M-052, and liposome) as well as the excipient composition (acyl chain length and saturation; PEGylated lipid:phospholipid ratio; and presence of antioxidant, tonicity, or viscosity agents) to maximize desired immunogenicity characteristics while maintaining physicochemical stability. This DOE/desirability index approach led to the identification of a lead candidate composition that demonstrated immune response durability and protective efficacy in the mouse model, as well as an assessment of the impact of each active vaccine formulation component on protection. Thus, we demonstrate that both GLA and 3M-052 are required for statistically significant protective efficacy. We also show that immunogenicity and efficacy results differ in female *vs* male mice, and the differences appear to be at least partly associated with adjuvant formulation composition.

## Introduction

Vaccine formulations often consist of multiple components including antigens, one or more immunomodulatory molecules, and formulation excipients. For example, GSK’s highly effective Shingrix^®^ vaccine for the prevention of shingles consists of a recombinant antigen and a combination adjuvant system consisting of a TLR4 ligand (MPL^®^) and a saponin (QS-21) in a liposomal formulation. Moreover, immunology assays enable a wide range of parameters to be monitored, each of which may be more or less dependent on the individual vaccine components. Despite this complexity, efficient dose and formulation optimization approaches employing tools such as Design of Experiments (DOE) or desirability functions are only rarely reported in vaccine development (1–3). Nevertheless, such approaches offer the benefit of objective multifactorial analysis while reducing the number of experimental subjects, such as animals, and the number of tests that would otherwise be required. Here, we employ DOE and desirability function approaches to optimize an *E. histolytica* vaccine candidate formulation consisting of a recombinant antigen (LecA) and a liposomal adjuvant formulation containing a synthetic TLR4 ligand (GLA) and a synthetic TLR7/8 ligand (3M-052).

Amebiasis is an enteric disease caused by infection with the *E. histolytica* parasite. Substantial morbidity is associated with amebiasis, particularly among children in endemic countries (4). Nevertheless, to our knowledge, no vaccine candidate has progressed to clinical testing to date. Encouragingly, protection from amebiasis in humans has been associated with mucosal IgA and IFNγ production from PBMCs (5, 6); in addition, IL-17A production from splenocytes was associated with protection in a mouse challenge model (7). Using enhancement of these immune readouts as objectives, we previously reported the proof-of-concept development of an intranasally administered vaccine antigen (LecA) adjuvanted with the combination adjuvant GLA-3M-052-liposomes, which resulted in enhanced fecal IgA, serum IgG, a mixed IFNγ and IL17A cellular immune response, and protective efficacy in immunized mice (8, 9). We now report the optimization of the dosing and excipient composition of this adjuvanted vaccine candidate using comprehensive stability, immunogenicity, durability, and efficacy data within a DOE and desirability function framework.

## Methods

### Materials

LecA antigen was manufactured by TECHLAB, Inc. (Blacksburg, VA) as described (10) and stored in phosphate buffered saline at 5°C; LecA endotoxin content was 0.03 EU/µg as measured by the limulus amebocyte lysate assay. Glucopyranosyl lipid adjuvant (GLA) was obtained from Avanti Polar Lipids (Alabaster, AL). 1,2-dipalmitoyl-sn-glycero-3-phosphocholine (DPPC), was obtained from Lipoid LLC (Newark, NJ), Corden Pharma (Liestal, Switzerland), and NOF Corporation (Tokyo, Japan). 1,2-dimyristoyl-sn-glycero-3-phosphocholine (DMPC), 1,2-dioleoyl-sn-glycero-3-phosphocholine (DOPC), and 1,2-distearoyl-sn-glycero-3-phosphocholine (DSPC) were obtained from Lipoid LLC. 1,2-distearoyl-sn-glycero-3-phosphoethanolamine-N-[methoxy(polyethylene glycol)-2000] (DSPE-PEG2000) was obtained from Corden Pharma, Lipoid LLC, NOF Corporation, and Nanocs Inc. (NY, NY). 1,2-dioleoyl-sn-glycero-3-phosphoethanolamine-N-[methoxy(polyethylene glycol)-2000] (DOPE-PEG2000) was obtained from Avanti Polar Lipids. 1,2-dimyristoyl-sn-glycero-3-phosphoethanolamine-N-[methoxy(polyethylene glycol)-2000] (DMPE-PEG2000), and 1,2-dipalmitoyl-sn-glycero-3-phosphoethanolamine-N-[methoxy(polyethylene glycol)-2000] (DPPE-PEG2000) were obtained from Corden Pharma. 3M-052 was provided courtesy of 3M Drug Delivery Systems (St. Paul, MN). Cholesterol was obtained from Corden Pharma, Sigma (St. Louis, MO), and Wilshire Technologies (Princeton, NJ). Ammonium phosphate monobasic and ammonium phosphate dibasic were purchased from J.T. Baker (San Francisco, CA). Microcrystalline cellulose/carboxymethylcellulose sodium (VIVAPUR^®^ MCG 811 P) was obtained from JRS Pharma (Patterson, NY). Glycerol and α-tocopherol were purchased from Spectrum Chemical (Gardena, CA).

### Preparation of Adjuvant Formulations

Batches of adjuvant were formulated at a 70-125-ml scale. GLA, 3M-052, phospholipid (DMPC, DPPC, DSPC, or DOPC), PEGylated lipid (DSPE-PEG2000, DPPE-PEG2000, DMPE-PEG2000, DOPE-PEG2000), cholesterol, and α-tocopherol (where indicated) were weighed and transferred to a 100-ml round bottom flask. Ten ml chloroform were added to the flask to dissolve the components, then the flask was placed on a rotary evaporator and the chloroform was evaporated to leave a thin film. The flask was left under vacuum overnight to assure complete solvent removal. Twenty-five mM ammonium phosphate with or without glycerol as indicated, pH=5.8, was added to the flask and the flask was placed in a 60°C water bath for 30 minutes to warm up. After warming, sonication was initiated and continued for 1 hour at 60°C to create multilamellar liposomes. The liposomes were then processed on a model LM20 microfluidizer (Microfluidics Corp.) for at least 5 passes at 18,000-30,000 psi. After microfluidization, the resulting liposomes were sterile filtered and filled into 3-ml serum vials with 1.2 ml/vial. The above procedure was adapted to make a high viscosity liposome batch by manufacturing *via* high shear mixing an aqueous phase containing ammonium phosphate buffer and microcrystalline cellulose/carboxymethylcellulose sodium, and then adding 25 ml of the aqueous phase to 50 ml of a 3x concentrated liposome formulation to achieve the target component concentrations as indicated. All liposomes were mixed 1:1 by volume with LecA/saline prior to immunization.

### Physicochemical Characterization of Adjuvant Formulation

Particle mean hydrodynamic diameter (Z-Ave_d_) and Polydispersity Index (PdI) were measured by Dynamic Light Scattering (DLS) on a Malvern Zetasizer Nano-ZS or -S (Worcestershire, UK), using a ZEN0023 Quartz flow cell and a NanoSampler for high-throughput handling, or a plastic cuvette. Nine measurements were obtained from a single sample preparation in 1.5 ml-autosampler vials. Samples were prepared by diluting formulation 1:100 in ultrapure (18.2 MΩ) water and subsequently vortexing for approximately five seconds.

All formulations were visually inspected and observations recorded prior to analysis at each time point. Samples were assessed for conformance with a homogeneous, translucent liquid formulation. Evidence of phase separation, large particulates/growth, or discoloration was monitored. pH was measured at each time point using an Accumet AB150 pH meter with a PerpHecT Ross Combination Micro Electrode. A 3-point calibration was performed using standards at pH 4.00, 7.00, and 10.00 prior to reading samples.

For analysis of GLA content, 50 µl of formulation were combined with 950 µl mobile phase B (1:2 [v/v] methanol:chloroform with 20 mM ammonium acetate and 1% acetic acid) in 1.5-ml glass vials. For each formulation, three separate vials were prepared. All samples were injected on a Waters (Milford, MA) XBridge C_18_ (5 µm, 4.6 x 250 mm) column at 30°C attached to an Agilent Model 1100 HPLC (Santa Clara, CA). A gradient consisting of mobile phase A (75:15:10 [v:v:v] methanol:chloroform:water with 20 mM ammonium acetate and 1% acetic acid) and mobile phase B was employed over 25 minutes at a flow rate of 1 ml/min. Detection was performed by an Thermo Fisher Scientific (Waltham, MA) Corona Charged Aerosol Detector (CAD). Quantitation was performed using a GLA standard at 0.05 µg/µl and varying inject volume to create a 9 point standard curve in mobile phase B.

The concentration of 3M-052 was determined by reverse-phase HPLC using an Agilent 1100 Series HPLC system (Santa Clara, CA) with UV/Vis diode array detector (DAD). The HPLC method consisted of first diluting the formulation at 1:20 or 1:5 volume ratio, depending on target concentration, in isopropanol containing 0.5% trifluoroacetic acid and then eluting the sample on a Zorbax Bonus-RP C14 Amide column (4.6 × 150 mm, 3.5 µm) at 45 °C with a 1 ml/min flow rate and the stop time of 30 minutes. An injection volume of 25 µl was used and a gradient mobile phase consisted of Mobile Phase A (0.1% trifluoroacetic acid in water), Mobile Phase B (methanol), and Mobile Phase C (isopropanol) as follows: initial (85% A, 15% B), 2.5 min (60% A, 40% B), 17.5 min (5% A, 40% B, 55% C), 22.0 min (5% A, 40% B, 55% C), 22.5 min (85% A, 15% B). A calibration curve was constructed for each run by injecting a 0.05 mg/ml 3M-052 standard at varied injection volumes and used to determine the concentration of 3M-052 from absorbance peak area at 321 nm.

Lipid excipient (phospholipid, PEGylated phospholipid, cholesterol) concentrations were determined by HPLC-CAD. The HPLC method utilized a 4.6 x 150 mm, 5 micron Zorbax Eclipse XDB-C18 column (Agilent Technologies). The mobile phase consisted of HPLC grade methanol and water (60:40) with 0.1% TFA on channel A and HPLC grade methanol and chloroform (60:40) with 0.1% TFA on channel B. A gradient was used starting at 60% B and ramping to 100% B over 45 minutes. Flow rate was controlled at 0.7 ml/min. The column thermostat was set to 25°C during operation. Standards for each lipid were prepared at concentrations of 100 µg/ml, 50 µg/ml, 25 µg/ml, 12.5 µg/ml, 6.25 µg/ml and 3.25 µg/ml. The liposomes were diluted 1:100 in chloroform:methanol (1:1) and compared to the standards to determine concentration. In selected formulations, Vitamin E was quantitated using the same method used for lipid excipients except that detection was performed by UV absorbance at 295 nm and samples were diluted 1:10 in chloroform:methanol (1:1). Vitamin E standards were prepared at concentrations of 20 µg/ml, 10 µg/ml, 5 µg/ml, 2.5 µg/ml, and 1.25 µg/ml.

Cryo-transmission electron microscopy was performed by NanoImaging Services, Inc. Samples were imaged undiluted by applying 3-µl drop on cleaned grid consisting of holey carbon films on 400-mesh copper grid, blotting with filter paper, and immediate vitrification in liquid ethane. Imaging was performed on an FEI Technai T12 electron microscope at 120 keV with FEI Eagle 4k x 4k CCD camera. The cryostage maintained the grid below -170°C. Images were acquired at 52,000x (0.21 nm/pixel) using electron doses of ~10-25 e^-^/Å^2^.

### Physicochemical Compatibility of Antigen and Adjuvant After Mixing

Short-term (≤24 h) physicochemical compatibility of the antigen-adjuvant mixture was evaluated by monitoring visual appearance, particle size, antigen primary structure by SDS-PAGE, retention of functionality by ELISA, and antigen-liposome association by ultracentrifugation. Analysis was performed immediately after mixing and 4 to 24 hours after mixing, with mixtures stored at 5°C. Two separate mixing studies were conducted for each batch of adjuvant: one focusing on adjuvant stability (visual appearance and particle size analysis) and the other focusing on antigen stability (SDS-PAGE, ELISA, and ultracentrifugation). The adjuvant-focused mixing study analysis was performed immediately after mixing and 4 to 24 hours after mixing, with mixtures stored at 5°C. The antigen-focused mixing study required three separate mixes corresponding to each of the three timepoints (time zero, 4 hours, and 24 hours) such that analysis for all timepoints could be performed at the same time. The antigen was first diluted in saline to 1.0 mg/ml, and subsequently mixed in 1:1 volume with the liposomal adjuvant formulation. For visual appearance samples were assessed for conformance with a homogeneous, translucent liquid formulation by monitoring potential phase separation, large particulates/growth, or discoloration. For particle size and polydispersity index, samples were diluted 5 μL into 495 μL of Milli-Q water into a disposable polystyrene cuvette, mixed by vortexing, then analyzed immediately on a Malvern Zetasizer-S or -ZS. For SDS-PAGE samples were diluted 10 μL into 30 μL of 4X NuPAGE LDS sample buffer, with 1.25% β-mercaptoethanol added, and incubated at 90-100°C for 15 minutes. Samples containing 1 μg of LecA were run at 180 V for 60 min in Life Technologies Novex WedgeWell Tris-Glycine 4–20% acrylamide, 1.0mm Tris-glycine precast gel cassettes using Novex Tris-glycine SDS running buffer after which they were fixed twice with 50% MeOH, 7% Acetic Acid for 30 minutes each then stained overnight using Life Technologies SYPRO Ruby stain. The following day, gels were washed with 10% MeOH, 7% Acetic Acid for 30 minutes, rinsed with with MilliQ water three times for 5 minutes then imaged using the ChemiDoc Imaging System (BioRad). Densitometry was performed using ImageLab (BioRad). ELISA was performed using the *E. Histolytica II* ELISA kit from TechLab (catalog #T5017). Samples were first diluted 10,000-fold in ELISA Dilution Buffer, followed by two 1:1 serial dilutions, in duplicate, for a total of 3 dilutions. A standard was generated by diluting the LecA antigen stock 14,625-fold to 80 ng/ml in Dilution Buffer, followed by a subsequent 3:4 dilution to generate 60 ng/ml. The 80 ng/ml standard was then serially diluted four times 1:1, and then 60 ng/ml standard diluted three times 1:1 for a total of 9 dilutions. The assay was performed according to the manufacturer’s instructions. A 5-parameter curve fit was calculated by SoftMax Pro software (Molecular Devices) to interpolate data points for the samples. Association of antigen with adjuvant was assessed by centrifuging the mixture at 160,000 x g for 1 h and assaying the supernatant by LecA ELISA as described above. Values were compared to supernatant from centrifuged LecA solution in the absence of adjuvant formulation.

### Immunizations

Male and female CBA/J mice 4-6 weeks old were purchased from The Jackson Laboratory, and allowed to acclimatize for 5 days at the vivarium prior to first immunization. In the dose and excipient optimization experiments that employed a DOE and/or desirability index approach, each experimental group consisted of 6 mice (3 male and 3 female) except for the antigen alone group in the first experiment which consisted of 5 mice (3 male and 2 female) since 1 female mouse was euthanized due to weight loss after the first immunization. In the immunogenicity durability study, each experimental group consisted of 8 mice (4 male and 4 female). In the protective efficacy studies, each experimental group consisted of 24 mice (12 male and 12 female). In all studies, mice were immunized on Days 0, 14, and 28 by intranasal delivery in 20 μL total volume (10 µl per nare) under anesthesia (**Supplementary Figure 1**). Due to the size of the dose and excipient optimization immunogenicity and lead candidate challenge experiments, immunizations occurred on two different days, with half of the animals in each group being immunized on one day and the other half of the animals being immunized on the next day. LecA and adjuvant formulation were mixed just prior to immunizations, with immunizations occurring within a 2-hour window after vaccine preparation. All formulations remained on wet ice until administration. Following each immunization animals were monitored for signs of adverse reactions including mortality, lethargy, weight loss and immunization site reactions.

### Sample Collection and Tissue Harvest

Mice were euthanized four weeks after the third immunization to collect plasma, bone marrow cells and splenocytes. On the day of tissue harvest, up to 500 μL (minimum 200 μL) of peripheral blood was collected into plasma separator tubes from axillary vessels under terminal anesthesia. Plasma was isolated *via* centrifugation and stored at -20°C until analysis for LecA-specific plasma antibody titers. Stool samples were collected three weeks after the third immunization and stool supernatants were prepared by vortexing stool pellets in PBS containing protease inhibitors, particulate matter was removed by centrifugation for 10 min at 900xg at 4°C, following which the supernatant was removed and centrifuged for 10 min at 15,800xg at 4°C, and supernatants were stored at - 20°C until analysis by ELISA. Stool and blood (tail vein puncture) were collected from 10 mice randomly before the first immunization to be used as a pre-immunization control baseline. Splenocytes were counted after RBC lysis and a total of 2 x 10^5^ splenocytes/well in 200 µL were stimulated with LecA at 50 µg/mL or left unstimulated in a 96 well U-bottom plate for 3 days at 37°C, 5% CO_2_. Supernatants were banked at -80°C. Bone marrow was collected from femurs at harvest and processed immediately for ELISpot analysis.

### Antibody ELISA

Antibody titers were measured using ELISA. High binding ELISA plates were coated with 0.5 µg/well recombinant LecA (TechLab lot# 71103) in 0.1 M bicarbonate buffer (pH 9.6) and blocked with 1% BSA-PBS. Following washes in PBS/Tween20, serially diluted plasma or stool samples were added. Plates were washed and anti-mouse IgG1-HRP, IgG2a-HRP, IgG-HRP or IgA-HRP followed by peroxidase substrate were added to the plates. Optical densities (OD) were read at 450 nm. Endpoint titers were interpolated using a cutoff OD of 0.5 and sigmoidal dose response (variable slope) least squares curve fits. Selected high-magnitude groups were diluted further to obtain measurable endpoint titers. Titration curves with R^2^ < 0.95 were visually assessed for removal of unambiguous outliers.

### Cytokine Bead Array (Luminex^®^)

Supernatants were analyzed for secreted cytokines (IFNγ and IL-17A) by R&D Systems Luminex° assays according to the manufacturer’s instructions. Signal from splenocytes stimulated with PMA-ionomycin served as a positive control and signal from blank well containing medium alone served as a negative control.

### ELISpot

Bone marrow cells were seeded for each mouse starting at 1 x 10^6^/100 µL plus three more 3-fold dilutions to 0.2 µg/well LecA pre-coated ELISPOT plates. Plates were incubated in a 37°C incubator with 5% CO_2_ for 3-5 hours, washed and anti-mouse IgG/IgA-HRP added. Plates were then incubated overnight at 4°C. AEC substrate solution was added for up-to 15 min and reaction was stopped by washing under running distilled water. Plates were dried in the dark for a minimum of 2 days and spots counted using an ELISPOT reader. Reported values were averaged from two of the dilutions. Reference serum from LecA immunized mice served as a positive control. Reference serum from unimmunized mice served as a negative control.

### Culture Conditions and Challenge Experiments

Protective efficacy experiments were conducted as described previously (8). Briefly, immunized mice were challenged intracecally 4 weeks after the final immunization with 2x10^6^trophozoites in 150 μl medium following laparotomy. Mice were euthanized a week after the challenge. Cecal contents were suspended in 1 ml PBS, with 300 μl cultured anaerobically in TYI-S-33 broth at 37°C for 5 days and 200 μl used for antigen load ELISA. TechLab E His II ELISA kit was used as per manufacturer’s instructions to detect presence of LecA antigen in cecal contents and to calculate infection rate. For quantitative antigen load evaluation using TechLab E His II kit, a standard curve was prepared using LecA. Parasite burden was also measured by quantitative PCR. In short, DNA was extracted from 200 µl cecal contents using QIAamp Fast DNA Stool Mini Kit (Qiagen). All samples were bead beaten for 2 min prior to DNA extraction. A standard curve was prepared from trophozoites. Primers, TaqMan probe and reaction conditions were as described previously (11). Vaccine efficacies were calculated as described (8).

### Design of Experiments (DOE) Methodology

The experimental design and randomization for the dose optimization DOE was performed using JMP v11.2 software, and modeling was performed using Design Expert v12 software to predict compositions that would best satisfy the desired immune profile from the entire experimental space. Since immunizations occurred on two consecutive days due to experiment size, the experimental groups were divided in half (with male and female mice included in each half), and animals were individually identified to ensure that each animal received subsequent immunizations on the appropriate day and following the randomized order indicated in **Supplementary Table 3**. Geometric means of each readout for each group of mice were employed as input for the model. To enable calculation of the geometric means for each group of mice, zero values were arbitrarily set to 0.5x the limit of detection for all readouts. Main, second order, third order and/or quadratic effects were modeled using partial least squares fitting. The resultant equations were evaluated for predictive power based on the Fisher F test (to determine if variation in response is dependent on the experimental conditions), the Lack of Fit F-value, the R^2^ calculations (including agreement between adjusted R^2^ and predicted R^2^) and residuals distribution to determine the variance explained by the model, and the actual performance of the test points compared to the model prediction. The top 25 predicted optimal responses were averaged to identify the center and range of the optimal composition.

### Statistical Analysis

Statistical analyses were performed using DesignExpert v12 and GraphPad Prism v8/v9 software. As indicated in the figure captions, immunogenicity readouts were log-transformed and analyzed by one-way or two-way ANOVA. As described in the text, alternative transformations were employed for selected readouts where recommended by DesignExpert v12 software during DOE analysis. Proportions of infected and uninfected mice from challenge trials were analyzed using two-sided Fisher’s exact test with or without Holm-Sidak’s correction for multiple comparisons as indicated in the table footnotes. Antigen load by ELISA or qPCR were analyzed using the non-parametric Kruskal-Wallis test with Dunn’s correction for multiple comparisons. P-values of less than 0.05 were considered statistically significant.

### Ethics Statement

All animal studies were conducted in strict accordance with the Guide for the Care and Use of Laboratory Animals (8th edition) of the National Institutes of Health. The protocol was approved by the International Animal Care and Use Committee at the University of Virginia (Protocol #4126; PHS Assurance #A3245-01). All surgeries were performed under ketamine/xylazine anesthesia; analgesics and supportive care were given to facilitate the well-being of the research animals.

## Results

Thirty-two separate batches of liposomal adjuvant formulations containing different compositions were manufactured to support mouse immunogenicity studies. The physicochemical characterization of each adjuvant formulation batch is described in **Supplementary Table 1**. In addition, selected adjuvant batches representing a range of compositions were mixed with the LecA antigen to determine physicochemical compatibility between antigen and adjuvant. Characterization of antigen and adjuvant characteristics following mixing indicated acceptable antigen-adjuvant compatibility for at least 24 h when stored at 5°C (**Supplementary Table 2**), thus supporting the point-of-care mixing immunization approach employed in the mouse studies. Although limited increase in liposome diameter occurred after mixing the LecA antigen with selected adjuvant formulations, the diameter remained <150 nm for all mixtures tested. Any association between antigen and adjuvant appeared in general to be weak since the majority of LecA remained in the supernatant when the liposomes were pelleted by centrifugation (**Supplementary Table 2**).

Two separate immunogenicity experiments were conducted based on DOE and/or desirability index methodology adapted from a previous report (3). The first immunogenicity experiment focused on optimization of vaccine formulation component doses (LecA antigen, GLA, 3M-052, phospholipid) using a central composite DOE. The second immunogenicity experiment was designed to refine excipient composition (lipid acyl chain structure, PEGylated lipid:phospholipid molar ratio, and presence of additional agents for tonicity, antioxidant, or viscosity control) using a desirability function approach. Each immunogenicity experiment and its results are described separately below. To inform the modeling for both studies, a weighted matrix was created based on previous mouse and human studies regarding correlates of protection from amebiasis, and various immunogenicity readouts were ranked, with 5 representing the greatest importance and 1 representing the least importance (**Table 1**). For example, IFN-γ and stool IgA have been shown to be associated with protection from *E. histolytica in* humans and are thus assigned the highest weight value. In addition, IL-17A and IFN-γ are correlated with protection in the mouse model and anti-LecA IgA inhibits adherence of trophozoites to mammalian cells *in vitro* (7, 10, 12). Although serum IgG subclass titers have not been correlated with protection, we hypothesized that the greater functionality of the IgG2a subclass may confer some protective benefit, whereas IgG1 would be less beneficial. Finally, we hypothesized that the durability of the immune response should be indicated by the frequency of bone-marrow resident LecA-specific antibody-secreting plasma cells (ASCs).

**Table 1:** Immune response desirability weighting.

Biological sample	Assay type	Assay target	Weight (1-5, 5 being most important)	Function	Rationale
Spleen	Cytokine bead array	IFN-γ	5	Maximize	IFNγ correlates with protection in humans and mice (6, 12)
Stool	ELISA	IgA	5	Maximize	Fecal IgA correlates with protection in humans (5)
Spleen	Cytokine bead array	IL-17A	4	Maximize	Depletion of IL-17A increases susceptibility in mice (7)
Bone marrow	ELISpot	IgA	3	Maximize	Plasma cells are necessary for durable IgA mediated immunity (13)
Bone marrow	ELISpot	IgG	2	Maximize	Plasma cells are necessary for durable IgG mediated immunity (13)
Blood	ELISA	IgG	2	Maximize	Serum IgG antibody titers are indicative of systemic immunogenicity (14)
Blood	ELISA	IgG2a/IgG1 ratio	2	Maximize	IgG2a/IgG1 ratio correlates with Th1 immunity (15)

To identify the experimental compositions that produced the best combination of immune responses matching the desired immune profile, response data were log-transformed and normalized by assigning a fractional desirability score of 0-1 for each immune response with 0 defined as the minimum response observed - (0.01)*(response range) and 1 defined as the maximum observed + (0.01)*(response range). The fractional desirability score, also called the desirability index (d_i_), was assigned based on the minimum and maximum log-transformed values observed across all formulations; for example, for readouts where maximal response was desirable, the log-transformed minimum antibody response – (0.01)*(response range) units (*x*) observed across all groups was subtracted from the log-transformed antibody titer (*y*) of the relevant experimental group, and the result would be divided by the difference between *x* and the log-transformed maximum antibody titer + (0.01)*(response range) units (*z*) observed across all groups: d_i_ = (*y*-*x*)/(*z*-*x*). For readouts where minimal response is desirable, the equation is as follows: d_i_ = (*z-y*)/(*z-x*). A weighted composite desirability score was then calculated using the equation $D=\sqrt[{p}]{{d_{1}}^{w_{1}}\times {d_{2}}^{w_{2}}\times \ldots \times {d_{n}}^{w_{n}}}$ where d_i_ = partial desirability attributed to the ith response (i=1; 2;…; n), w_i_ = weighting attributed to the ith response and $p=\Sigma_{1}^{n}w_{i}$ (16).

### Dose Optimization DOE

We employed a central composite face-centered response surface design to optimize the doses of the four main components (LecA, GLA, 3M-052, phospholipid). This design requires three levels of each of the four continuous factors. Twenty-seven experimental groups (including 3 centerpoint runs) were designed and randomized. Based on previous experience with dosing in the mouse model, we defined the high, mid, and low dose values for each of the components as follows: LecA (10, 1, 0.1 μg), GLA (10, 1, 0.1 μg), 3M-052 (4, 0.4, 0.04 μg), and phospholipid (216, 72, 24 μg). The actual measured content values for each manufactured formulation were within 32% of the target values and can be found in **Supplementary Table 1**. The doses of other lipid excipients (cholesterol and PEGylated lipid) varied according to phospholipid dose to maintain a constant ratio between lipid excipients (molar ratio of phospholipid:PEGylated lipid:cholesterol 12.2:1.0:7.1). Two additional experimental groups were added to the experiment: one was a test group described previously (8) and here termed the ‘proof-of-concept composition’ to validate the model’s predictive power (by employing intermediate doses of 5 μg LecA, 5 μg GLA, 2 μg 3M-052, and 72 μg phospholipid), and one was an antigen-alone control group (10 μg LecA with no TLR ligands or lipid excipients). The experimental group details are shown in **Supplementary Table 3**. Mice were immunized three times at two-week intervals, and sample collection occurred four weeks after the final immunization (**Supplementary Figure 1**).

Following the first immunization there was a transient weight loss in several groups, but all groups had recovered their mean pre-immunization weight by day 7 with the exception of the group receiving antigen alone (**Supplementary Figure 2**). Likewise, transient weight loss followed by weight gain in many groups was repeated after the second and third immunizations although the magnitude of weight loss was decreased. These data suggest that there is no severe or sustained impact on the animals’ health after immunization with the antigen and adjuvant dose ranges evaluated here.

**Figure 1:**
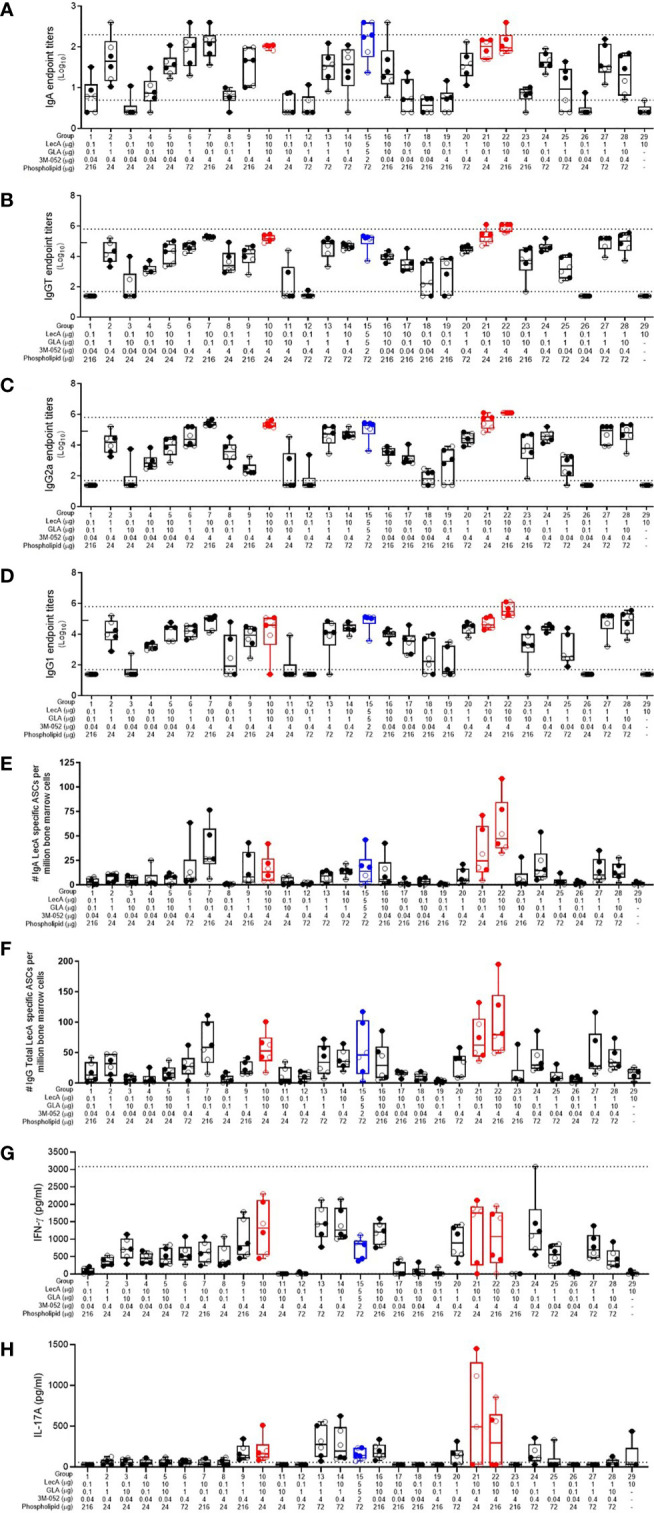
Antibody and cellular immune responses elicited in mice 4 weeks following the last immunization in the dose optimization DOE. **(A)** stool LecA IgA titers, **(B)** plasma LecA IgG total titers, **(C)** plasma LecA IgG2a titers, **(D)** plasma LecA IgG1 titers, **(E)** LecA-specific IgA-secreting bone marrow cells, **(F)** LecA-specific IgG-secreting bone marrow cells, **(G)** LecA-specific IFNγ secretion by splenocytes, and **(H)** LecA-specific IL-17A secretion by splenocytes. For all panels, the proof-of-concept group (#15) is shown in blue and the overall top 3 responding groups (21, 10, and 22) from the combined data analysis are shown in red. Females are represented by closed circles and males by open circles. The box-whisker plots represents the median values (bars), the 25^th^-75^th^ percentiles (boxes), and the minimum and maximum values (whiskers), with all points shown. Dotted lines indicate the upper and lower limits of quantitation; responses outside of these ranges were arbitrarily set to 2x (serum antibody endpoint titers) or 1x (cytokine responses) the upper limit and 0.5x the lower limit. For **(B-D)**, the tick mark on the y-axis at 4.9 represents the initial upper limit; selected groups (7, 10, 15, 20, 21, 22, and 28) were then further diluted to the higher upper limit shown by the dotted line due to high magnitude titers.

Component dosing had a strong impact on the magnitude and quality of immune responses (**Figure 1**). Antigen and adjuvant dosing clearly influenced the LecA-specific stool IgA response (**Figure 1A**). Notably, all of the groups that contained the low antigen dose (0.1 μg LecA) showed little or no IgA response regardless of adjuvant component dosing. Likewise, the antigen-alone control (10 μg LecA without adjuvant) did not produce detectable LecA-specific stool IgA titers. The highest responses were generated in groups with higher antigen (1-10 µg) and TLR ligand doses (1-10 µg GLA and/or 0.4-4 µg 3M-052). Plasma LecA-specific total IgG (IgGT), IgG1 and IgG2a titers followed a similar trend as the stool IgA titers except that overall response magnitudes were greater, with some response evident in the low dose LecA groups when high doses of TLR ligand(s) were present (**Figures 1B–D**).

The number of LecA-specific IgA- and IgG-secreting bone marrow plasma cells were measured using ELISpot (**Figures 1E, F**). The antigen alone control group elicited minimal numbers of LecA-specific plasma cells, again indicating the importance of adjuvant inclusion. As with the antibody responses, IgG-secreting bone marrow plasma cells were more frequent than IgA-secreting bone marrow plasma cells. The highest number of antibody-secreting bone marrow plasma cells were elicited in the experimental groups immunized with both the high dose of LecA antigen (10 µg) and the high dose of 3M-052 (4 µg). Regarding cellular immune responses, compositions that resulted in enhancement of IFNγ production from antigen-stimulated splenocytes also tended to increase IL-17A production, although to a lesser extent (**Figures 1G, H**). Only low levels of IL-2, IL-5, and TNFα were detected (data not shown).

Overall, there were substantial differences in the antibody and cellular responses between the various groups depending on component dosing, as expected. The sex of the animal had a much smaller, but still significant, impact on the antibody responses, with females generally generating higher responses than males; however, no significant differences due to sex were evident in the cytokine responses (**Supplementary Table 4**). Thus, the percent of total response variation attributable to sex for the measured immune responses varied from 0.0 – 2.5% whereas the immunization composition (component dosing) contributed 54.6 – 85.1% of the response variation.

Employing the desirability index function as described above, the partial desirability for each immune response and the overall weighted composite desirability for the experimental groups were calculated using the log-transformed geometric means of all readouts, with the IgG2a/IgG1 ratio representing the ratio of the respective log-transformed values. Experimental groups 21, 10, and 22 produced the highest desirability responses (**Table 2**). Interestingly, these three groups were immunized with the high doses of LecA (10 µg) and 3M-052 (4 µg), whereas GLA and phospholipid doses were low or high, suggesting that LecA and 3M-052 were the most important components to induce the desired immune response. The experimental variability is apparent by comparing the performance of the three replicate center points (groups 6, 20, and 27), where response magnitude aligned well across readouts except for IL-17A where no response was detected in group 27, thus affecting overall desirability score.

**Table 2:** Desirability index scores of each experimental group in the first immunogenicity experiment based on measured immune responses.

Group #	Component Dosing (LecA, GLA,3M-052, DPPC)*	IFN-γ	Fecal IgA	IL-17A	Bone Marrow ASC IgA	Bone Marrow ASC IgG	serum IgGT	serum IgG2a/IgG1 ratio	Overall Score (Female)	Overall Score (Male)	Overall Score (Male and Female)
**21**	H, L, H, L	0.818	0.874	0.962	0.815	0.916	0.856	0.683	0.800	0.811	0.852
**10**	H, H, H, L	0.953	0.902	0.836	0.668	0.836	0.831	0.840	0.871	0.754	0.849
**22**	H, H, H, H	0.783	0.943	0.723	0.990	0.990	0.990	0.579	0.802	0.786	0.841
**15**	Proof-of-concept	0.868	0.990	0.755	0.593	0.729	0.787	0.507	0.828	0.643	0.773
**14**	H, M, M, M	0.988	0.585	0.934	0.694	0.759	0.715	0.557	0.746	0.709	0.754
**13**	M, M, H, M	0.990	0.617	0.990	0.536	0.696	0.693	0.711	0.771	0.671	0.753
**24**	M, L, M, M	0.957	0.695	0.673	0.726	0.746	0.711	0.516	0.701	0.713	0.732
**16**	H, H, L, H	0.954	0.600	0.826	0.549	0.627	0.566	0.311	0.673	0.576	0.654
**7**	H, L, H, H	0.826	0.935	0.199	0.851	0.858	0.843	0.629	0.659	0.595	0.653
**20**	M, M, M, M	0.888	0.648	0.575	0.528	0.665	0.681	0.474	0.651	0.592	0.648
**6**	M, M, M, M	0.816	0.858	0.249	0.617	0.620	0.707	0.562	0.650	0.502	0.604
**2**	M, M, M, L	0.743	0.715	0.348	0.537	0.637	0.630	0.455	0.437	0.650	0.577
**28**	M, H, M, M	0.762	0.496	0.279	0.644	0.763	0.761	0.456	0.580	0.456	0.545
**5**	H, H, L, L	0.784	0.639	0.269	0.410	0.529	0.626	0.362	0.492	0.449	0.507
**9**	M, M, M, H	0.908	0.644	0.796	0.605	0.625	0.574	0.010	0.499	0.472	0.490
**25**	M, M, L, M	0.805	0.300	0.197	0.327	0.418	0.404	0.296	0.261	0.263	0.369
**4**	H, L, L, L	0.782	0.266	0.271	0.387	0.203	0.387	0.351	0.380	0.327	0.366
**27**	M, M, M, M	0.871	0.687	0.010	0.647	0.761	0.766	0.435	0.362	0.283	0.336
**8**	L, L, H, L	0.774	0.193	0.193	0.073	0.173	0.491	0.990	0.281	0.286	0.285
**3**	L, H, L, L	0.855	0.038	0.285	0.377	0.224	0.143	0.592	0.298	0.137	0.238
**17**	H, L, L, H	0.255	0.205	0.010	0.106	0.516	0.471	0.338	0.174	0.079	0.141
**1**	L, L, L, H	0.394	0.210	0.010	0.222	0.446	0.010	0.460	0.123	0.116	0.125
**18**	L, H, L, H	0.211	0.076	0.010	0.298	0.395	0.235	0.125	0.134	0.055	0.105
**23**	L, H, H, H	0.010	0.223	0.010	0.403	0.336	0.490	0.631	0.092	0.080	0.086
**29**	LecA alone (H)	0.112	0.010	0.259	0.196	0.443	0.010	0.460	0.131	0.039	0.085
**12**	L, M, M, M	0.081	0.069	0.010	0.185	0.377	0.024	0.764	0.088	0.044	0.075
**19**	L, L, H, H	0.114	0.154	0.010	0.010	0.010	0.317	0.837	0.040	0.087	0.060
**11**	L, H, H, L	0.010	0.067	0.010	0.311	0.230	0.174	0.738	0.045	0.043	0.057
**26**	L, L, L, L	0.083	0.022	0.010	0.178	0.139	0.010	0.460	0.084	0.018	0.048

*H, High; M, Mid; L, Low. For LecA and GLA; H,10 µg; M,1 µg; L,0.1 µg. For 3M-052; H,4 µg; M,0.4 µg; L,0.04 µg. For DPPC; H,216 µg; M,72 µg; L,24 µg. Color scale, higher desirability index values are light blue; lower desirability index values are dark blue.

To predict an optimal composition that would best satisfy the desired immune profile from the entire experimental space rather than just the tested points, DOE software (DesignExpert v12) was employed to model main effects, second order, third order and quadratic effects using partial least squares fitting with up to 4 factors (i.e. dose of LecA, GLA, 3M052, and phospholipid). Only the 27 experimental groups comprising the central composite face-centered design were employed in the DOE prediction analysis (i.e. the antigen-alone and the proof-of-concept test groups were not included). The input in the DOE model consisted of the geometric means of each readout for each experimental group. Log-transformed values for the serum antibody, bone marrow ASC, and IL-17A readouts were employed. However, a square-root transformation was employed for the IFN-γ readout to avoid a significant lack of fit.

**Figure 2:**
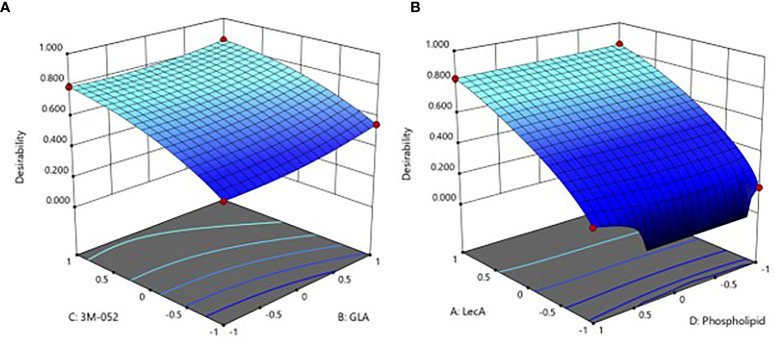
Predicted immune response desirability as a continuous function of **(A)** GLA and 3M-052 doses, or **(B)** LecA and phospholipid doses. The axes values of +1, 0, and -1 correspond to the high, mid, and low component doses (see footnote to **Table 2** for actual values). The plot in **(A)** represents the effect of changing GLA and 3M-052 dose when phospholipid dose is set to the lowest level (-1) and LecA dose is set to the highest level (+1). The plot in **(B)** represents the effect of changing phospholipid and LecA dose when GLA and 3M-052 doses are set to the highest level (+1). Color scale: higher desirability index values are light blue, lower desirability index values are dark blue.

Following model selection and refinement for each readout as described in the Methods section, the resultant DOE model equations were found to have statistically significant predictive power, although the ASC and serum antibody readouts generally demonstrated stronger model fit performance compared to the cytokine readouts (**Supplementary Table 5**). Furthermore, LecA and 3M-052 doses appeared to be the most influential factors in the model. The integrated desirability response surface was continuous for all 4 factors (**Figure 2**), and the top 25 predicted optimal responses were averaged to identify the center and range of the optimal composition (**Table 3**). Moreover, the validity of the model was confirmed by the responses elicited by the proof-of-concept composition (experimental group #15), which generally aligned well with the predicted value ranges generated by the model (**Supplementary Table 6**).

**Table 3:** Predicted optimal component dosing ranges to maximize the desired immune response*.

	LecA (µg/dose)	GLA (µg/dose)	3M-052 (µg/dose)	Phospholipid (µg/dose)
average	9.93	9.86	3.77	29.40
min	9.27	8.67	2.50	24.00
max	10.00	10.00	4.00	50.44
Doses selected for subsequent studies	10	10	4	34

*Desirability scores among the top 25 predicted optimal responses represented in this table ranged from 0.837 to 0.849 with an average desirability score of 0.843.

Thus, the DOE analysis resulted in selection of a predicted optimal composition consisting of 10 µg LecA, 10 µg GLA, 4 µg 3M-052, and 29 µg phospholipid. Since the predicted optimal LecA, GLA, and 3M-052 doses are at the limit of the explored experimental space, it is possible that higher doses would be still more potent. Nevertheless, the upper dose limits were selected based on practical or safety considerations (manufacturability, stock concentration availability, injection volume constraints, or potential for reactogenicity). Selecting a relatively low phospholipid dose was also anticipated to be beneficial due to adjuvant physicochemical stability characterization data, which indicated that higher phospholipid doses resulted in decreased pH and phospholipid content stability, possibly due to insufficient buffering capacity (**Supplementary Figure 3**). However, a slightly higher phospholipid dose of 34 µg (rather than 29 µg, see **Table 3**) was selected for subsequent studies since manufacturing challenges including increased particle size and lower recovery of liposome components were evident in some batches manufactured at the lower end of the phospholipid dose range (see **Supplementary Table 1**).

### Excipient Composition Immunogenicity Study

The next immunogenicity experiment was designed to (1) confirm the predictive utility of the model developed in the DOE described above, (2) evaluate the impact of additional excipients (α-tocopherol as an antioxidant, glycerol as a tonicity agent, and microcrystalline cellulose/carboxymethylcellulose sodium as a viscosity agent), and (3) identify the optimal liposomal phospholipid acyl chain length, acyl chain saturation, and phospholipid:PEGylated lipid molar ratio, resulting in a total of 20 experimental groups. The experimental group details are shown in **Supplementary Table 7** and measured content values for each manufactured adjuvant formulation can be found in **Supplementary Table 1**. Mice were immunized three times at two-week intervals, and sample collection occurred four weeks after the final immunization (**Supplementary Figure 1**).

Similar to the first immunogenicity experiment described above, there was a minor and transient weight loss in many groups but all groups had recovered their mean pre-immunization weight by day 7 (**Supplementary Figure 4**). These data suggest that there is no severe or sustained impact on the animals’ health after immunization for the excipient compositions evaluated.

**Figure 3:**
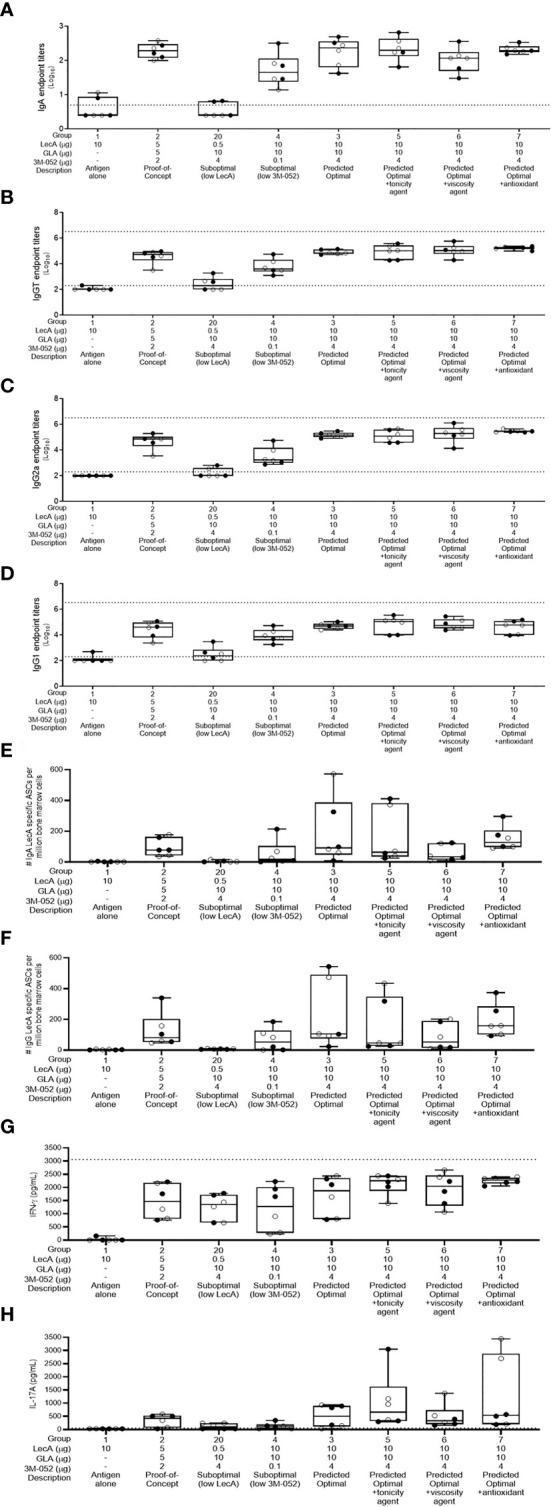
Antibody and cellular immune responses elicited in mice 4 weeks following the last immunization in groups 1-7 and group 20 of the excipient composition immunogenicity study (see **Supplementary Table 4**). **(A)** stool LecA IgA titers, **(B)** plasma LecA IgG total titers, **(C)** plasma LecA IgG2a titers, **(D)** plasma LecA IgG1 titers, **(E)** LecA-specific IgA-secreting bone marrow cells, **(F)** LecA-specific IgG-secreting bone marrow cells, **(G)** LecA-specific IFNγ secretion by splenocytes, and **(H)** LecA-specific IL-17A secretion by splenocytes. For all panels, females are represented by closed circles and males by open circles. The box-whisker plots represent the median values (bars), the 25^th^-75^th^ percentiles (boxes), and the minimum and maximum values (whiskers), with all points shown. Dotted lines indicate the upper and lower limits of quantitation; responses below the lower limit were arbitrarily set to 0.5x the lower limit, and no responses were detected above the upper limit.

For easier interpretation, we have plotted the immune responses from the experimental groups related to model prediction confirmation and the impact of additional excipients in **Figure 3**, whereas the experimental groups related to lipid acyl chain structure and ratios (where little or no difference in response was apparent) are plotted separately in **Supplementary Figure 5**. All experimental groups were analyzed using the desirability index criteria described above to calculate overall immunogenicity desirability scores (**Table 4**). The predictive power of the DOE model developed from the first immunogenicity experiment was verified regarding the magnitude and quality of immune responses elicited by the predicted optimal and suboptimal compositions compared to the control groups (i.e. antigen alone and proof-of-concept composition, see **Figure 3**). In general, the predicted optimal composition tended to generate the highest serum antibody titers, antibody-secreting bone marrow plasma cells, and cytokine production from splenocytes, whereas the suboptimal compositions demonstrated substantially reduced performance. Furthermore, addition of glycerol or α-tocopherol did not impair antibody or cytokine responses. In contrast, addition of microcrystalline cellulose/carboxymethylcellulose sodium appeared to reduce stool IgA titer and antibody-secreting bone marrow plasma cells. Desirability index analysis of immunogenicity responses (**Table 4**) confirmed the potency of the predicted optimal composition and indicated potential benefit from glycerol or α-tocopherol inclusion, while microcrystalline cellulose/carboxymethylcellulose inclusion resulted in a reduced desirability score.

**Table 4:** Desirability index scores of each experimental group in the second immunogenicity experiment based on immunogenicity and adjuvant physicochemical stability readouts.

Group #	Description*	IFN-γ	Fecal IgA	IL-17A	Bone Marrow ASC IgA	Bone Marrow ASC IgG	serum IgGT	serum IgG2a/IgG1 ratio	Immuno-genicity Score (female)	Immuno-genicity Score (male)	Overall Immuno-genicity Score	Overall Adjuvant Stability Score**	Combined Overall Score
**7**	PO + antioxidant	0.984	0.981	0.861	0.919	0.914	0.822	0.990	0.896	0.920	0.932	0.886	0.909
**5**	PO + tonicity agent	0.971	0.990	0.868	0.839	0.752	0.746	0.590	0.854	0.833	0.859	0.683	0.766
**15**	DPPC, Mid ratio	0.932	0.874	0.871	0.924	0.937	0.912	0.493	0.903	0.778	0.857	0.669	0.757
**3**	PO	0.912	0.936	0.621	0.845	0.868	0.740	0.736	0.834	0.757	0.815	0.687	0.749
**19**	DSPC, Mid ratio	0.963	0.715	0.873	0.861	0.919	0.823	0.745	0.941	0.703	0.840	0.598	0.709
**12**	DMPC, Mid ratio	0.978	0.762	0.844	0.694	0.691	0.990	0.433	0.765	0.771	0.781	0.628	0.700
**17**	DOPC, High ratio	0.975	0.908	0.886	0.721	0.795	0.904	0.393	0.720	0.874	0.818	0.591	0.696
**11**	DMPC, Low ratio	0.952	0.858	0.681	0.782	0.867	0.858	0.614	0.844	0.749	0.810	0.562	0.675
**16**	DSPC, High ratio	0.987	0.842	0.990	0.832	0.850	0.878	0.497	0.817	0.877	0.859	0.526	0.672
**8**	DPPC, Low ratio	0.990	0.863	0.818	0.653	0.689	0.829	0.863	0.687	0.898	0.830	0.450	0.611
**9**	DOPC, Mid ratio	0.973	0.658	0.695	0.649	0.786	0.737	0.633	0.788	0.666	0.738	0.504	0.610
**14**	DSPC, Low ratio	0.971	0.971	0.935	0.990	0.990	0.936	0.376	0.931	0.826	0.889	0.369	0.573
**10**	DOPC, Low ratio	0.974	0.796	0.755	0.831	0.855	0.801	0.523	0.792	0.775	0.804	0.241	0.440
**2**	POC	0.890	0.960	0.558	0.815	0.801	0.651	0.575	0.780	0.725	0.766	0.231	0.420
**13**	DPPC, High ratio	0.966	0.962	0.921	0.981	0.963	0.946	0.128	0.891	0.697	0.803	0.195	0.396
**18**	DMPC, High ratio	0.967	0.719	0.694	0.870	0.866	0.814	0.559	0.735	0.804	0.785	0.137	0.328
**20**	PSO (less LecA)	0.862	0.010	0.281	0.135	0.247	0.104	0.027	0.077	0.161	0.116	0.669	0.279
**4**	PSO (less 3M052)	0.809	0.655	0.314	0.495	0.462	0.452	0.010	0.570	0.305	0.379	0.108	0.203
**6**	PO + viscosity agent	0.947	0.814	0.699	0.663	0.648	0.777	0.674	0.722	0.781	0.766	N/A	N/A
**1**	LecA alone	0.010	0.041	0.010	0.010	0.010	0.010	0.195	0.013	0.023	0.017	N/A	N/A

LecA dose was 10 µg for all groups except for the POC formulation (5 µg) and the PSO-less LecA (0.5 µg). *PO, Predicted Optimal formulation from dose optimization DOE; PSO, Predicted Sub-Optimal formulations from dose optimization DOE; POC, Proof-of-Concept formulation (same as group #15 from the dose optimization DOE); DMPC, DPPC, DSPC, DOPC refers to acyl chain structure of liposomal lipids; High, Mid, Low refers to phospholipid: PEGylated lipid ratios. See **Supplementary Table 7** for additional formulation composition details. **See **Supplementary Table 8** for detailed adjuvant stability results. Color scale: higher desirability index values are light blue, lower desirability index values are dark blue.

The effects of lipid acyl chain length and saturation were more subtle, with little difference between groups in most readouts although saturated acyl chain compositions appeared to perform slightly better overall compared to unsaturated acyl chain compositions at the same phospholipid:PEGylated lipid ratios and acyl chain length (**Supplementary Figure 5** and **Table 4**). Likewise, there did not appear to be a consistent trend regarding the effects of phospholipid:PEGylated lipid ratio variation, although the low or high ratios appeared to perform slightly better than the middle ratios with the exception of the DPPC (16:0/16:0) compositions where the middle ratio was the top performer (**Supplementary Figure 5** and **Table 4**). In contrast, there were more noticeable impacts of these factors on formulation physicochemical stability (**Supplementary Tables 8, 9**). Moreover, major stability impacts were evident in the compositions containing additional excipients. Since stability is a key consideration in the development of pharmaceutically acceptable adjuvant formulations, the desirability index analysis was expanded to include stability data collected over 3-6 months on formulation vials stored at 40°C or 25°C. Changes in adjuvant and excipient chemical content, pH, and particle size characteristics were influenced by the excipient acyl chain structure and ratios employed (**Supplementary Tables 8, 9**). By combining the stability desirability index score with the immunogenicity desirability index score on an equal weight basis, a combined desirability index score was calculated and employed to rank the formulations in the second immunogenicity experiment (**Table 4**).

As in the first immunogenicity experiment, the sex of the animal had a much smaller, but still significant, impact on immune responses in the second immunogenicity experiment; however, in contrast to the first immunogenicity experiment, the significant differences due to sex in the second immunogenicity experiment were evident in the cytokine responses rather than the antibody responses, with higher IL-17A response in males but higher IFNγ response in females (**Supplementary Table 10**). The percent of total response variation attributable to sex for the measured immune responses varied from 0.0 – 2.0% whereas the formulation composition contributed 53.8 - 89.0% of the response variation.

### Lead Candidate Selection and Characterization

**Figure 4:**
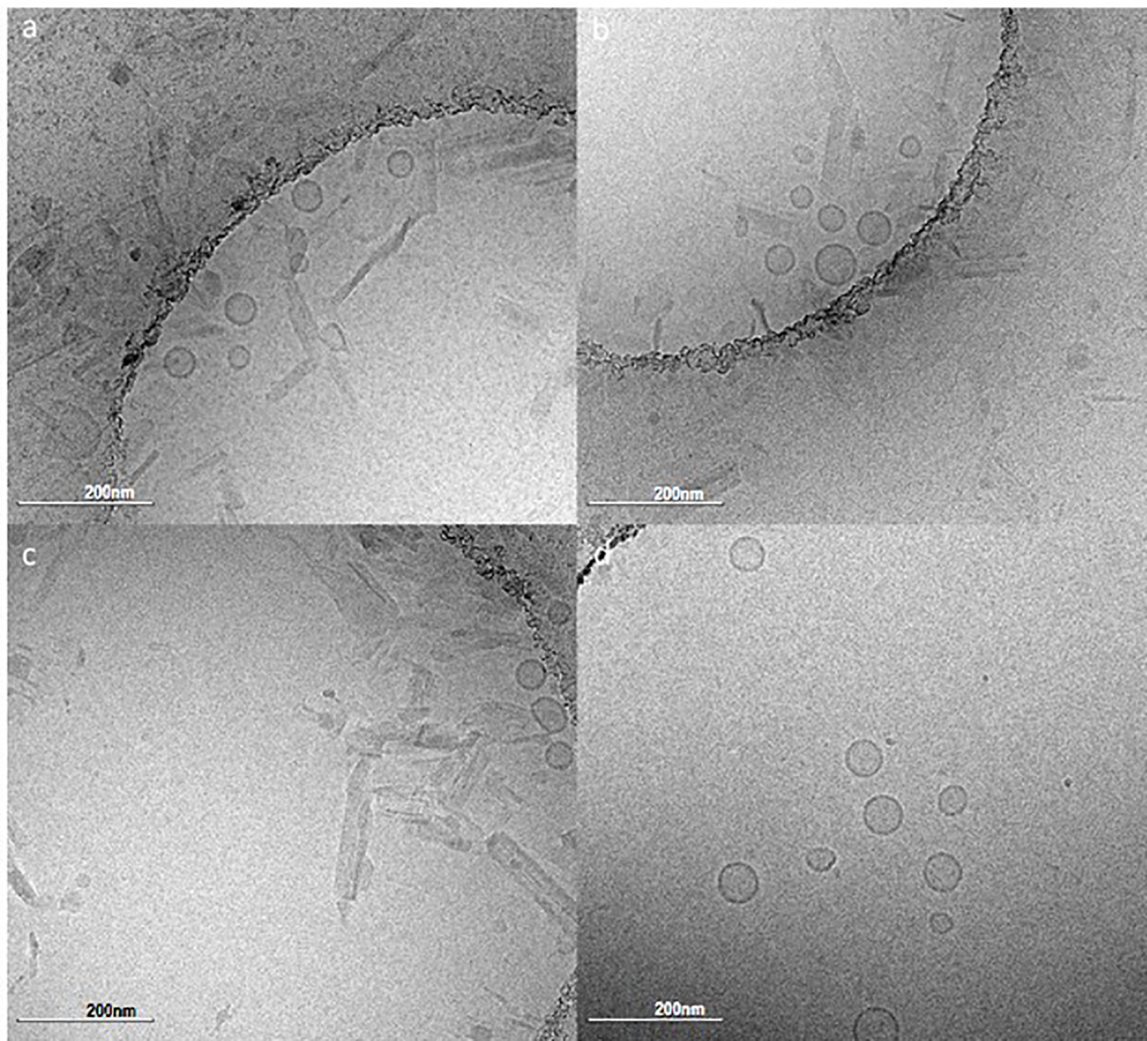
Cryo-transmission electron microscopy monographs of the lead candidate adjuvant formulations and the proof-of-concept adjuvant formulation. **(A)** PS composition, **(B)** PP composition, **(C)** SS composition, **(D)** Proof-of-concept composition. Refer to **Table 5** for composition details. Scale bar represents 200 nm.

Based on the analysis of immunogenicity and stability criteria described above, three lead candidate adjuvant formulations (denoted as PS, PP, and SS to represent the acyl chain composition of the lipid components, i.e. PS is comprised of a palmitoyl acyl chain for the primary phospholipid and a stearoyl acyl chain for the PEGylated phospholipid) were selected for further evaluation. The selected lead candidates correspond to groups 7, 15, and 19, respectively, from **Table 4**, except that antioxidant was added to the compositions from groups 15 and 19 to generate the lead candidates. Although it was also a top performer as shown in **Table 4**, the composition represented by Group 5 was not selected as a lead candidate after preliminary evaluation indicated the tonicity agent would have negative impacts on performance of a nasal delivery device that was planned for use in future studies (data not shown). The composition and physicochemical characterization of the three lead candidate adjuvant formulations are shown in **Table 5**; the composition of the proof-of-concept adjuvant formulation is included for comparison. All three lead candidates were formulated with the antioxidant α-tocopherol for enhanced stability (**Table 5**). Formulation morphology of the three lead candidate adjuvant formulations was assessed by cryo-transmission electron microscopy and compared to the morphology of a new batch of the proof-of-concept formulation manufactured at the same time as the lead candidate compositions (**Figure 4**). The images indicated the expected morphology for the proof-of-concept formulation based on previous experience, consisting of unilamellar vesicles <100 nm in diameter with some disk-like structures. The three lead candidate adjuvant formulations also contained unilamellar vesicles generally smaller than 100 nm as expected; however, extensive linear striated structures of irregular lengths up to ~500 nm were also present. These structures appeared most prevalent in lead candidate #3 ‘SS’. The different morphology apparent in the lead candidates compared to the proof-of-concept formulation may be due to their increased TLR ligand:phospholipid ratio which could change the preferred packing orientation and curvature of the lipid structures; it is also possible that the addition of α-tocopherol played a role. Despite the novel morphology of the lead candidate formulations, dynamic light scattering data over time indicate that the compositions are physically stable (data not shown).

**Table 5:** Lead candidate adjuvant formulation characteristics.

Lead Candidate Name	GLA* (mg/ml)	3M-052* (mg/ml)	Primary phospho lipid* (mg/ml)	PEG-ylated phospho lipid* (mg/ml)	Cholesterol* (mg/ml)	α-tocopherol* (mg/ml)	Buffer (ammonium phosphate)	pH	Particle diameter (Z-ave, nm)	Size Polydisp-ersity Index (PdI)
PS	1.04 +/- 0.01	0.36 +/- 0.00	3.10 +/- 0.10 (DPPC)	0.92 +/- 0.02 (DSPE-PEG2000)	0.86 +/- 0.03	0.06 +/- 0.00	25 mM	5.83	81.4 +/- 1.4	0.169 +/- 0.014
PP	1.04 +/- 0.01	0.39 +/- 0.01	3.21 +/- 0.09 (DPPC)	0.92 +/- 0.05 (DPPE-PEG2000)	0.90 +/- 0.04	0.06 +/- 0.00	25 mM	5.85	90.8 +/- 2.8	0.218 +/- 0.013
SS	1.02 +/- 0.01	0.36 +/- 0.01	2.89 +/- 0.11 (DSPC)	0.84 +/- 0.03 (DSPE-PEG2000)	0.80 +/- 0.02	0.06 +/- 0.00	25 mM	5.75	85.4 +/- 1.2	0.217 +/- 0.005
Proof-of-concept	0.52 +/- 0.01	0.19 +/- 0.00	6.06 +/- 0.22	2.00 +/- 0.12	1.88 +/- 0.07	–	25 mM	5.76	67.3 +/- 1.6	0.217 +/- 0.008

*Dose amounts (µg) correspond to 0.01*concentration (i.e. 1 mg/ml GLA in the adjuvant formulation results in delivery of 10 µg of GLA after mixing with antigen/diluent and delivering 20 µl total volume to the nares).

### Immune Response Durability

**Figure 5:**
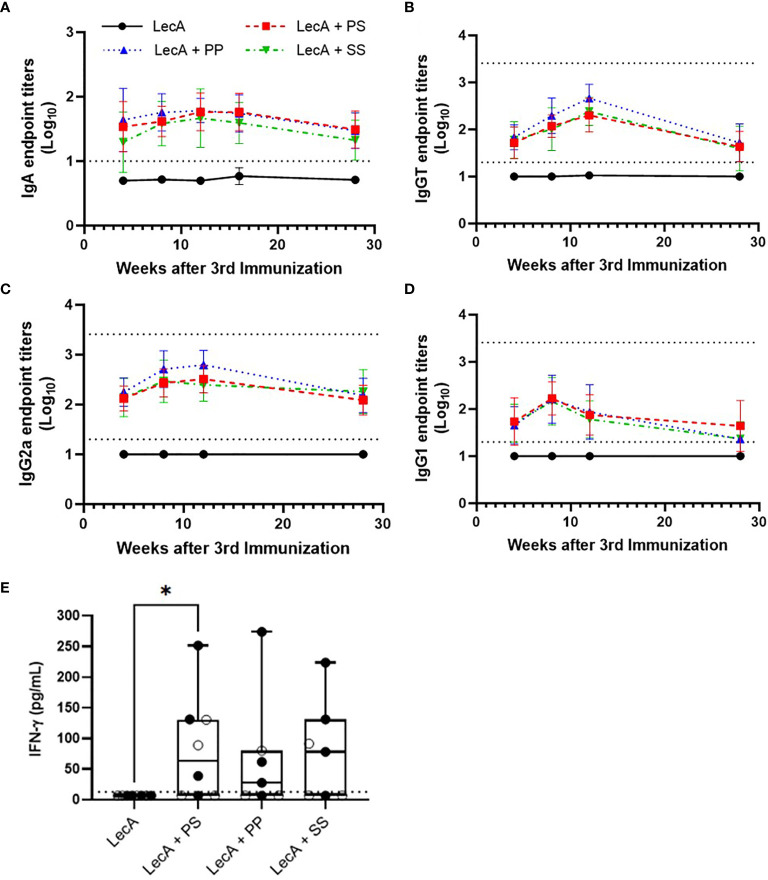
Long-term antibody and cellular immune responses elicited in mice immunized with antigen alone or combined with lead candidate adjuvant formulations at 4, 8, 12, 16, and/or 28 weeks after the last immunization as shown. **(A)** stool LecA IgA titers, **(B)** plasma LecA IgG total titers, **(C)** plasma LecA IgG2a titers, **(D)** plasma LecA IgG1 titers, **(E)** LecA-specific IFNγ secretion by splenocytes at 28 weeks post-final immunization. For panel **(E)**, females are represented by closed circles and males by open circles. For panels **(A–D)**, bars represent means and error bars represent standard deviations. Dotted lines indicate the upper and lower limits of quantitation. Responses below the lower limit were arbitrarily set to 0.5x the lower limit, and no responses were detected above the upper limit. All antibody titers in experimental groups receiving adjuvant were significantly higher (p < 0.05) than the antigen alone group at all time points, except for IgG1 at 28 weeks post-final immunization for the PP and SS groups, as analyzed by two-way ANOVA with the Dunnett correction for multiple comparisons. For panel **(E)** the box-whisker plot represents the median values (bars), the 25^th^-75^th^ percentiles (boxes), and the minimum and maximum values (whiskers), with all points shown; *p < 0.05 (analysis by one-way ANOVA on log-transformed values). LecA dose for all groups was 10 µg.

A long-term immunogenicity experiment involving the three lead candidate adjuvant formulations was conducted to evaluate immune response durability. Following the same immunization regime employed for the immunogenicity experiments described above (**Supplementary Figure 1**), mice were immunized with LecA antigen alone (10 µg) or combined with each of the three lead candidate adjuvant formulations. Sera and stool samples were collected at intervals up to 28 weeks post-final immunization (**Supplementary Figure 1**). To evaluate long-term cytokine response, splenocytes were also collected at 28 weeks post-final immunization for cytokine analysis (intermediate time points were not possible since this readout requires a terminal endpoint). Stool and serum antibody responses peaked at 8-12 weeks post-final immunization but persisted at detectable levels through 28 weeks for adjuvanted groups (**Figure 5**). Likewise, production of IFNγ from stimulated splenocytes was detectable for all three adjuvanted groups at 28 weeks, with LecA + PS achieving statistical significance compared to LecA alone. In contrast, no IL-17A was found.

Taken together, the immunogenicity durability study indicated detectable antigen-specific antibody and cellular immune responses even 28 weeks after final immunization; however, there was little or no difference in performance between the three lead candidate adjuvant formulations. Regarding differences attributable to sex, stool IgA responses in female mice were significantly higher compared to male mice in LecA + PP and LecA + SS groups; in addition, serum IgGT and serum IgG2a responses in female mice were significantly higher compared to male mice in the LecA + SS group (**Supplementary Table 11**). Thus, sex appeared to have the greatest impact overall in the formulation containing the longest saturated acyl chains (SS).

### Protective Efficacy

**Figure 6:**
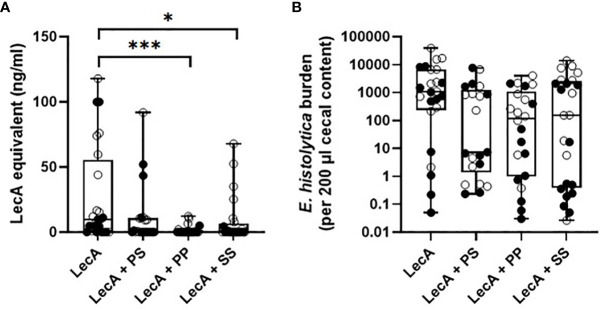
Protective efficacy in mice intracecally challenged 4 weeks after the last of three immunizations with antigen alone or combined with lead candidate adjuvant formulations. **(A)** cecal antigen load measured by ELISA, **(B)** cecal antigen load measured by qPCR. For both panels, females are represented by closed circles and males by open circles. The box-whisker plots represent the median values (bars), the 25^th^-75^th^ percentiles (boxes), and the minimum and maximum values (whiskers), with all points shown. ***p < 0.001, *p < 0.05 by Kruskal-Wallis test with Dunn’s correction for multiple comparisons. LecA dose for all groups was 10 µg.

The three lead candidate formulations (**Table 5**) were evaluated for protective efficacy in a mouse challenge model of amebic colitis. Mice were immunized three times at two-week intervals with LecA antigen alone or combined with each of the three lead candidate adjuvant formulations (**Supplementary Figure 1**). Four weeks following the final immunization, mice were cecally challenged with 2 x 10^6^ trophozoites. Cecal contents were then analyzed one week following the challenge. Protective efficacy was evaluated by cecal content culture for live ameba, ELISA, and qPCR (**Table 6** and **Figure 6**). Culture results demonstrated that 75% of the animals receiving LecA antigen alone were infected, whereas groups receiving LecA combined with adjuvant formulation had infection rates ranging from 27-48% depending on adjuvant formulation and readout (culture or ELISA). Thus, infection rates were reduced by 37-64% compared to the LecA antigen alone control, which is consistent with our previous report on the efficacy of the proof-of-concept composition evaluated on male mice (8). Moreover, quantitative ELISA revealed that mean antigen load was reduced by 59-94% in adjuvanted groups compared to antigen alone. Likewise, qPCR indicated reduced antigen load in the adjuvanted groups. Interestingly, female mice were significantly more likely to be protected from infection and have reduced antigen load compared to male mice. Indeed, combining the results for all males and females revealed a statistically significant impact of sex on protective efficacy by Fisher’s exact test (p<0.05), with 28 of 46 males but only 16 of 45 females infected as measured by cecal content ELISA (**Table 6**). Overall, lead candidate ‘PP’ appeared to be the top performer for protective efficacy.

**Table 6:** Mouse infection rate as determined by cecal content ELISA and live ameba culture.

	LecA alone	LecA + Lead Adjuvant Formulation Candidate #1 ‘PS’	LecA + Lead Adjuvant Formulation Candidate #2 ‘PP’	LecA + Lead Adjuvant Formulation Candidate #3 ‘SS’
% infected (culture)	75.0	47.6	40.9*	45.8
% infected (ELISA)	75.0	47.6	27.3*,**	41.7*
# infected males (ELISA)	10 of 12	6 of 11	5 of 11	7 of 12
# infected females (ELISA)	8 of 12	4 of 10	1 of 11	3 of 12

LecA dose for all groups was 10 µg. *p < 0.05 by two-sided Fisher’s exact test without correction for multiple comparisons. **p < 0.01 by two-sided Fisher’s exact test with the Holm-Sidak correction for multiple comparisons.

**Figure 7:**
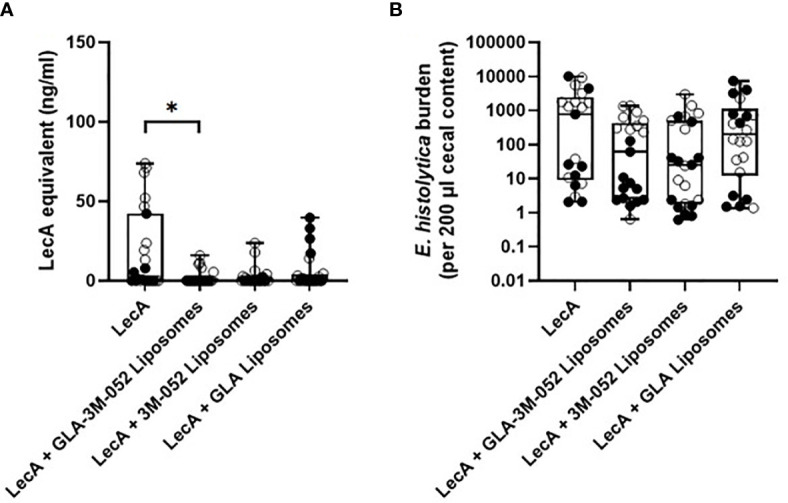
Protective efficacy in mice intracecally challenged 4 weeks after the last of three immunizations with antigen alone, with antigen and dual TLR ligand lead candidate adjuvant formulation, or with antigen and single TLR ligand adjuvant formulation. **(A)** cecal antigen load measured by ELISA, **(B)** cecal antigen load measured by qPCR. Due to space constraints, six mice were not represented on the qPCR analysis plate (2 females from LecA group, 2 males from LecA + GLA-3M-052 liposomes group, and 2 females from LecA + GLA liposomes group; all 6 mice were negative for infection by culture and ELISA). For both panels, females are represented by closed circles and males by open circles. The box-whisker plots represent the median values (bars), the 25^th^-75^th^ percentiles (boxes), and the minimum and maximum values (whiskers), with all points shown. *p < 0.05 by Kruskal-Wallis test with Dunn’s correction for multiple comparisons. LecA dose for all groups was 10 µg.

To determine the impact of each TLR ligand on protective efficacy, a second efficacy experiment was conducted following the same immunization and challenge regimen and readouts described above. Mice were immunized with LecA antigen alone or LecA with the ‘PP’ lead candidate adjuvant formulation containing both TLR ligands (**Table 5**) or a single TLR ligand (**Supplementary Table 12**). Protective efficacy was evaluated by cecal content culture for live ameba, ELISA, and qPCR (**Table 7** and **Figure 7**). An infection rate of 22-26% occurred in animals receiving the lead candidate formulation containing both TLR ligands, whereas higher infection rates occurred in animals receiving single TLR ligand formulations (35-50%) or LecA alone (65%), see **Table 7**. Quantitative ELISA revealed that mean antigen load was reduced by 88% in the dual TLR ligand formulation group, 86% in the 3M-052 liposome group, and 68% in the GLA liposome group compared to antigen alone. Similarly, qPCR indicated reduced antigen load in the adjuvanted groups. These results emphasize the impact on protective efficacy of including both TLR ligands in the lead candidate adjuvant formulation, since only the dual TLR ligand formulation achieved statistical significance compared to the antigen alone in the efficacy readouts. Furthermore, the trend toward improved efficacy with 3M-052 liposomes compared to GLA liposomes corroborates the DOE immunogenicity analysis that indicated that 3M-052 dose was a more influential factor than the GLA dose (**Figure 1** and **Supplementary Table 5**). Moreover, the infection rate results of the lead candidate and LecA alone control were consistent between both challenge experiments in which they were employed (**Tables 6**, **7**), emphasizing the reproducibility of the challenge model results. Another consistency between both efficacy studies is that more males than females were infected overall, but particularly in 3M-052-containing formulations (**Tables 6**, **7**). If the infection rate results from both challenge studies are combined (**Tables 6**, **7**), 51% of males and 20% of females were infected after immunization with LecA + GLA-3M-052 liposome or 3M-052 liposome formulations (p<0.05 by Fisher’s exact test). On the other hand, male mice immunized with LecA antigen alone or LecA + GLA-liposomes demonstrated an infection rate of 64% compared to 60% for females (not statistically significant).

**Table 7:** Impact of TLR ligands on mouse infection rate as determined by cecal content ELISA and live ameba culture.

	LecA alone	LecA + GLA-3M-052 Liposomes ‘PP’	LecA + 3M-052 Liposomes	LecA + GLA Liposomes
% infected (culture)	65.2	21.7*,**	34.8	50.0
% infected (ELISA)	65.2	26.1*,**	34.8	45.8
# males infected (ELISA)	8 of 12	5 of 12	6 of 11	5 of 12
# females infected (ELISA)	7 of 11	1 of 11	2 of 12	6 of 12

LecA dose for all groups was 10 µg. *p < 0.05 by two-sided Fisher’s exact test without correction for multiple comparisons. **p < 0.05 by two-sided Fisher’s exact test with the Holm-Sidak correction for multiple comparisons.

## Discussion

In previous work, we identified liposomes containing the synthetic TLR ligands GLA and 3M-052 as a promising adjuvant formulation to promote antigen-specific IFNγ/IL-17A-associated immunogenicity and protective efficacy in the amebic colitis mouse model (8, 9). Nevertheless, optimization of component doses (antigen, TLR ligands, and phospholipid) and excipient composition including phospholipid acyl chain structure and PEG density had not previously been attempted. Moreover, examination of the impact of sex on immune responses and protective efficacy had not been evaluated.

Employment of a DOE and desirability index framework allowed simultaneous evaluation of multiple factors while minimizing animal usage. The value of such an approach is maximized when the implications of the immunological (and stability) readouts are well understood. For instance, the association of stool IgA and PBMC IFNγ with protection from amebiasis in humans informed the weighting factors of the immunological readouts employed in the DOE and desirability index analysis here. Even when correlates of immunity are known, the weighting of the various readouts will always have some degree of subjectivity and may require refinement as more knowledge is gained (3). Another potential benefit of the DOE and desirability index approach is identification of candidates that elicit different immune response profiles. Such candidates could then be evaluated in challenge studies to determine how specific immune responses correlate to protective efficacy. Nevertheless, a limitation of the current study is that the splenocytes responsible for producing the IFNγ and IL-17A cytokine responses were not dissected to reveal the proportion of the cytokine response elicited by specific cell types such as CD4+ T cells, CD8+ T cells, or NK cells.

Regarding component dosing, it was shown that 10 µg LecA, 10 µg GLA and 4 µg 3M-052 maximized the desired immune profile (**Table 3**). These GLA and 3M-052 doses are within the ranges previously employed in parenteral immunization applications in mice (17, 18), indicating that intranasal delivery is efficient enough to generate potent immune responses with liposomal TLR ligands in combination with a recombinant protein antigen. Moreover, it is worth noting that the optimal doses of GLA and 3M-052 identified in the current study are within the range of doses currently employed in clinical trials for parenteral administration of these molecular adjuvants (19–22). While theoretically it might have been possible to predict that the maximum doses of antigen and TLR ligands would result in the highest magnitude immune responses, the DOE analysis allows visualization of the dosing window profiles for each component (**Figure 2**), enabling prediction of the impact of changing component doses if needed due to safety, manufacturability, or related considerations. For instance, compared to the impact of LecA and 3M-052 dosing, GLA and phospholipid dosing had reduced but still significant influence on the resulting immune response (**Figure 2**, **Table 7** and **Supplementary Table 5**). In addition, it appears that increasing TLR ligand density in liposomes (by reducing phospholipid dose) does not dramatically change immune response magnitude or quality at the dose ranges evaluated here, in contrast to results obtained with a parenteral HIV vaccine candidate liposome formulation containing GLA (23). However, it is difficult to compare results across studies due to differences in administration route, formulation composition, immunogen, etc. Indeed, reducing phospholipid dose in the present study resulted in a different particulate structure (**Figure 4**) and it is unclear whether such structures have different biological activities than traditional unilamellar liposomes when used to deliver TLR ligands.

Regarding excipient composition, changes in liposomal phospholipid acyl chain structure (length and saturation) and thus membrane fluidity have been shown to be important factors in the resulting immune response magnitude and quality (24, 25). Although the literature is not conclusive, it appears that in many cases saturated phospholipids are preferred for generating maximum Th1-type immune responses to liposome-associated vaccine antigens (24, 25). This finding is consistent with the present study, where saturated DSPC-based liposomes appeared to elicit slightly improved responses overall compared to unsaturated DOPC-based liposomes even though the LecA antigen appears not to be associated with the liposomes (**Table 4** and **Supplementary Figure 5**). However, it is problematic to make meaningful comparisons on this point since there are many other differentiating factors such as antigen encapsulation/association, liposome size, adjuvant structure, etc. Another excipient question of interest is density of PEGylation, which can affect liposome trafficking and cellular interactions (8). Only subtle immunological differences due to PEG density were apparent in the data reported here, although physicochemical stability impacts were more evident (**Table 4**, **Supplementary Table 9** and **Supplementary Figure 5**).

The most impactful excipient on enhancing liposome stability was inclusion of the antioxidant α-tocopherol, and there appeared to be a trend for improved immunogenicity responses as well (**Figure 3**, **Table 4**, and **Supplementary Table 9**). Oil-in-water emulsions containing α-tocopherol are known to exhibit potent vaccine adjuvant activity at high concentrations; indeed, the AS03 adjuvant has been included in pandemic influenza vaccines that have achieved licensure (26, 27). Nevertheless, it is important to point out that the dose of α-tocopherol employed in AS03 is >1,000-fold greater than the dose employed in the current report, and any adjuvant activity attributable to α-tocopherol was absent when tested at a low dose (100-fold less than the dose in AS03) in another oil-in-water emulsion (28). Thus, an adjuvant effect of α-tocopherol at the extremely low dose employed here would be unexpected and would require further investigation to verify. In any case, its antioxidant properties are essential for enhanced liposome stability.

Another excipient evaluated in the current study was the viscosity enhancer microcrystalline cellulose/carboxymethylcellulose sodium. Viscosity enhancers are expected to increase nasal residence time and therefore uptake into antigen-presenting cells, and some reports indicate a beneficial effect of viscosity enhancers in nasal vaccine preparations in various animal models (29, 30). However, it is difficult to untangle potential innate adjuvant effects of the viscosity enhancer itself aside from its effect on delivery kinetics. Viscosity may also affect anatomical deposition of nasal formulations, and excessive viscosity may actually result in lower nasal retention time (31). The concentration of viscosity enhancer employed in the current report was based on the excipient manufacturer’s recommendations and resulted in an estimated final vaccine formulation viscosity increase of an estimated ~300-fold compared to the same formulation without the viscosity enhancer (the actual viscosity was not measured due to material volume limitations). Nevertheless, the viscosity enhancer did not improve immune responses. Notably, a clinical trial of a nasal influenza vaccine with or without a microcrystalline cellulose/carboxymethylcellulose sodium-based viscosity enhancer showed no immunological benefit associated with the inclusion of the viscosity enhancer (32). Whether different viscosity enhancers or optimization of the concentration of viscosity enhancer would improve vaccine immune responses merits further evaluation. However, we note that inclusion of the viscosity enhancer in the present case presented an additional difficulty, which was interference with many of the physicochemical characterization methods used to assess the stability of the formulation due to the particulate nature of microcrystalline cellulose/carboxymethylcellulose sodium (**Supplementary Table 8**).

The generation of overall higher magnitude immune responses in female mice compared to males is consistent with recent reports (33). Surprisingly, phospholipid acyl chain structure appeared to play a role in this regard, with liposomes containing DSPC resulting in the greatest sex-based differences in the long-term immunogenicity experiments (**Supplementary Tables 10** and **11**). Since DSPC is a common lipid formulation component [including in the currently approved mRNA COVID-19 vaccines (34)], more investigation on this point is worthwhile. Moreover, future work focusing on the impact of sex on immunogenicity responses would benefit from increased numbers of males and females per group. Perhaps of even greater interest was the impact of sex and TLR ligand composition on protective efficacy since it appeared that the difference in protective efficacy between male and female mice may be associated with inclusion of the 3M-052 component (**Tables 6** and **7**, **Figures 6** and **7**). This finding is consistent with a report that female mice immunized with inactivated influenza vaccine had greater antibody response and protection against influenza challenge than male mice, and this result was found to be associated with greater TLR7 expression in the B cells of vaccinated female mice (33). Thus, the impact of biological sex on the efficacy of vaccines containing natural TLR7 ligands (such as inactivated viral vaccines) or synthetic TLR7 ligands such as 3M-052 may have important implications for vaccine design and evaluation. In any case, both GLA and 3M-052 are necessary for statistically significant protective efficacy in the mouse model of amebic colitis evaluated here, emphasizing the potential benefits of including multiple TLR ligands in adjuvant formulation design. Indeed, a multi-TLR ligand approach more closely represents the mechanisms triggered by effective live attenuated vaccines such as the yellow fever vaccine (35). Nevertheless, there are no existing adjuvant formulations in licensed vaccines that contain more than one TLR ligand. Moreover, given that TLR expression varies widely between animal models and humans (36, 37), it is important to determine the relevance of the findings identified here to other models.

## Conclusion

The complexity of vaccine adjuvant formulation and evaluation requires a robust experimental design methodology to meaningfully interrogate the effects of multiple factors on multiple immunological and physicochemical stability readouts. A DOE and desirability index approach was employed here, resulting in the optimization of a dual TLR ligand adjuvant formulation composition demonstrating strong immune response durability and protective efficacy in the mouse model of amebic colitis. Furthermore, the importance of inclusion of both TLR ligands (GLA and 3M-052) for protective efficacy was demonstrated. Moreover, biological sex was shown to be a significant factor in immunogenicity and efficacy responses. Together, these findings have resulted in a lead candidate vaccine adjuvant composition suitable for advanced preclinical development. The intranasal adjuvant platform developed here may have relevance for vaccine development against other enteric or respiratory pathogens.

## Data Availability Statement

The original contributions presented in the study are included in the article/**Supplementary Material**. Further inquiries can be directed to the corresponding author.

## Ethics Statement

The animal study was reviewed and approved by the Institutional Animal Care and Use Committee at the University of Virginia.

## Author Contributions

MA and MO: conceptualization, data curation, formal analysis, investigation, methodology, project administration, supervision, visualization, writing. RK, IL, EL, and JG: investigation, data curation, methodology, writing. SS and SL: data curation, project administration. AN and DO: investigation, data curation. MY, LF, MU, JL, and SB: investigation. HL: investigation, methodology, data curation. AK, PG, and SR: investigation, data curation, methodology. MT: resources (provision of 3M-052). KP: resources (provision of LecA), methodology. WP: conceptualization, methodology, project administration, supervision, funding acquisition. CF: conceptualization, data curation, formal analysis, investigation, methodology, project administration, supervision, visualization, writing, funding acquisition. All authors contributed to the article and approved the submitted version.

## Funding

This work was supported by federal funds from the National Institute of Allergy and Infectious Diseases (NIAID), National Institutes of Health (NIH), Department of Health and Human Services, under Contract HHSN272201800025C.

## Conflict of Interest

MT is an employee of 3M and 3M-052 is an asset of 3M’s. WP is a consultant for TechLab, Inc. and in addition receives royalties for amebiasis diagnostics that are donated in their entirety to the American Society of Tropical Medicine and Hygiene. KP is an employee of TechLab, Inc. and amebiasis diagnostics are an asset of TechLab’s. CF, RK, SS, HL, IL, EL, JG, SL, AK, and SR are employees of IDRI, which owns assets including patents and patent applications involving formulations of GLA and 3M-052 including what is represented in this article. MA, WP, CF, and SL are inventors on patent/patent application(s) involving the vaccine formulations represented in this article.

The remaining authors declare that the research was conducted in the absence of any commercial or financial relationships that could be construed as a potential conflict of interest.
