# Supplementary material for: Optimizing a Multi-Component Intranasal Entamoeba Histolytica Vaccine Formulation Using a Design of Experiments Strategy
Source: Front Immunol. 2021 Jun 25;12:683157. doi: 10.3389/fimmu.2021.683157 (PMC8268010; doi:10.3389/fimmu.2021.683157)
Supplement: Supplementary file 1 [file DataSheet_1.docx]

**Supplementary Table 1. Characterization of adjuvant formulations used in the mouse immunogenicity studies.**

| Batch# | Target GLA conc. (mg/ml) | Measured GLA conc. (mg/ml) | Target 3M-052 conc. (mg/ml) | Measured 3M-052 conc. (mg/ml) | Target phospholipid conc. (mg/ml) | Measured phospholipid conc. (mg/ml) | Particle Diameter (Z-ave, nm) | pH |
| --- | --- | --- | --- | --- | --- | --- | --- | --- |
| QH392 | 0.500 | 0.468 | 0.200 | 0.181 | 7.2 | 6.5 | 75 | 5.7 |
| QH407 | 0.010 | 0.010 | 0.004 | 0.004 | 21.6 | 19.7 | 56 | 5.7 |
| QH409 | 0.100 | 0.114 | 0.040 | 0.036 | 2.4 | 2.1 | 69 | 5.7 |
| QH410 | 1.000 | 0.964 | 0.004 | 0.004 | 2.4 | 2.4 | 70 | 5.7 |
| QH413 | 0.010 | 0.011 | 0.004 | 0.005 | 2.4 | 2.3 | 58 | 5.8 |
| QH416 | 0.100 | 0.112 | 0.040 | 0.040 | 7.2 | 6.9 | 56 | 5.8 |
| QH436 | 0.010 | 0.010 | 0.400 | 0.331 | 21.6 | 18.8 | 63 | 5.9 |
| QH439 | 0.010 | 0.011 | 0.400 | 0.275 | 2.4 | 2.1 | 111 | 5.8 |
| QH441 | 0.100 | 0.112 | 0.040 | 0.039 | 21.6 | 17.9 | 52 | 5.7 |
| QH443 | 1.000 | 0.925 | 0.400 | 0.392 | 2.4 | 2.1 | 86 | 5.8 |
| QH445 | 0.100 | 0.104 | 0.400 | 0.373 | 7.2 | 6.4 | 74 | 5.9 |
| QH448 | 1.000 | 0.942 | 0.004 | 0.004 | 21.6 | 19.5 | 65 | 5.7 |
| QH449 | 1.000 | 0.944 | 0.400 | 0.377 | 21.6 | 20.3 | 62 | 5.9 |
| QH450 | 0.010 | 0.013 | 0.040 | 0.043 | 7.2 | 7.4 | 56 | 5.8 |
| QH451 | 0.100 | 0.111 | 0.004 | 0.004 | 7.2 | 7.2 | 63 | 5.8 |
| QH521 | 1.000 | 0.865 | 0.400 | 0.315 | 3.6 | 2.8 | 115 | 5.8 |
| QH522 | 1.000 | 0.980 | 0.400 | 0.374 | 3.1 | 2.8 | 86 | 5.8 |
| QH523 | 1.000 | 0.994 | 0.400 | 0.365 | 3.6 | 3.5 | 76 | 5.8 |
| QH524 | 1.000 | 0.938 | 0.400 | 0.327 | 3.4 | 3.2 | 80 | 5.8 |
| QH525 | 1.000 | 0.998 | 0.400 | 0.329 | 3.4 | 3.1 | 77 | 5.8 |
| QH526* | 1.000 | -- | 0.400 | -- | 3.4 | -- | -- | 5.9 |
| QH527 | 1.000 | 0.965 | 0.400 | 0.319 | 3.4 | 3.3 | 83 | 5.8 |
| QH528 | 1.000 | 0.983 | 0.400 | 0.329 | 3.6 | 3.2 | 81 | 5.8 |
| QH529 | 1.000 | 1.006 | 0.400 | 0.339 | 3.6 | 3.2 | 76 | 5.8 |
| QH530 | 1.000 | 0.946 | 0.400 | 0.359 | 3.4 | 3.2 | 99 | 5.8 |
| QH531 | 1.000 | 0.909 | 0.400 | 0.354 | 3.4 | 3.1 | 83 | 5.8 |
| QH532 | 1.000 | 0.945 | 0.400 | 0.350 | 3.4 | 3.1 | 58 | 5.8 |
| QH536 | 1.000 | 0.876 | 0.400 | 0.353 | 3.1 | 2.8 | 91 | 5.8 |
| QH537 | 1.000 | 0.876 | 0.400 | 0.368 | 3.1 | 2.8 | 61 | 5.7 |
| QH539 | 1.000 | 0.846 | 0.400 | 0.347 | 3.6 | 3.3 | 116 | 5.8 |
| QH540 | 1.000 | 0.850 | 0.400 | 0.362 | 3.6 | 3.2 | 93 | 5.8 |
| QH542 | 1.000 | 0.887 | 0.010 | 0.009 | 3.4 | 3.0 | 73 | 5.7 |

*The viscosity agent in QH526 interfered with HPLC and particle size assays, preventing accurate data acquisition.

**Supplementary Table 2. Characterization of antigen-adjuvant compatibility following mixing.**

| Mixture Composition | Particle diameter after 4h at 5°C (Z-ave, nm) | Particle diameter after 24h at 5°C (Z-ave, nm) | % Change in SDS-PAGE LecA band density after 4h at 5°C | % Change in SDS-PAGE LecA band density after 24h at 5°C | % Change in LecA ELISA content after 4h at 5°C | % Change in LecA ELISA content after 24h at 5°C | % Liposome-associated LecA after 24h at 5°C |
| --- | --- | --- | --- | --- | --- | --- | --- |
| LecA+QH407 | 77 | 70 | -3% | -2% | 1% | 4% | -8% |
| LecA+QH410 | 96 | 101 | -5% | -2% | 2% | 5% | 7% |
| LecA+QH413 | 107 | 100 | 10% | 5% | -1% | -2% | 12% |
| LecA+QH416 | 80 | 94 | 3% | 4% | 18% | 14% | 11% |
| LecA+QH436 | 104 | 88 | -5% | 0% | -5% | 2% | -3% |
| LecA+QH439 | 103 | 143 | 1% | 2% | -2% | 1% | -2% |
| LecA+QH443 | 116 | 137 | 2% | 2% | -4% | -6% | 28% |
| LecA+QH448 | 80 | 74 | 3% | -4% | 3% | 13% | 5% |
| LecA+QH449 | 98 | 93 | 3% | 0% | -2% | 10% | -1% |
| LecA+QH521 | 132 | 135 | -4% | 0% | 0% | -2% | -8% |
| LecA+QH522 | 81 | 95 | 0% | 0% | 14% | 11% | 7% |
| LecA+QH523 | 78 | 83 | -1% | 4% | 4% | 14% | 3% |
| LecA+QH524 | 108 | 121 | -1% | -5% | -4% | -6% | 0% |
| LecA+QH525 | 106 | 120 | -4% | -1% | 3% | -7% | -3% |

Notes: No change in formulation visual appearance was apparent for any of the mixtures. LecA concentration for all mixtures was 0.1 mg/ml. Saline was used as diluent to achieve the target LecA concentration. Adjuvant formulation comprised half of the volume of all mixtures. Percent change was calculated with regards to reference LecA band (SDS-PAGE), T=0 mixture value (ELISA), or centrifuged LecA without liposome (association).

**Supplementary Table 3. Experimental groups for dose optimization DOE.**

| Group # | Target LecA dose (µg) | Target GLA dose (µg) | Target 3M-052 dose (µg) | Target Phospholipid (DPPC) dose (µg) | Adjuvant Batch# |
| --- | --- | --- | --- | --- | --- |
| 1 | 0.1 | 0.1 | 0.04 | 216 | QH407 |
| 2 | 1 | 1 | 0.40 | 24 | QH409 |
| 3 | 0.1 | 10 | 0.04 | 24 | QH410 |
| 4 | 10 | 0.1 | 0.04 | 24 | QH413 |
| 5 | 10 | 10 | 0.04 | 24 | QH410 |
| 6 | 1 | 1 | 0.40 | 72 | QH416 |
| 7 | 10 | 0.1 | 4.00 | 216 | QH436 |
| 8 | 0.1 | 0.1 | 4.00 | 24 | QH439 |
| 9 | 1 | 1 | 0.40 | 216 | QH441 |
| 10 | 10 | 10 | 4.00 | 24 | QH443 |
| 11 | 0.1 | 10 | 4.00 | 24 | QH443 |
| 12 | 0.1 | 1 | 0.40 | 72 | QH416 |
| 13 | 1 | 1 | 4.00 | 72 | QH445 |
| 14 | 10 | 1 | 0.40 | 72 | QH416 |
| 15 | 5 | 5 | 2.00 | 72 | QH392 |
| 16 | 10 | 10 | 0.04 | 216 | QH448 |
| 17 | 10 | 0.1 | 0.04 | 216 | QH407 |
| 18 | 0.1 | 10 | 0.04 | 216 | QH448 |
| 19 | 0.1 | 0.1 | 4.00 | 216 | QH436 |
| 20 | 1 | 1 | 0.40 | 72 | QH416 |
| 21 | 10 | 0.1 | 4.00 | 24 | QH439 |
| 22 | 10 | 10 | 4.00 | 216 | QH449 |
| 23 | 0.1 | 10 | 4.00 | 216 | QH449 |
| 24 | 1 | 0.1 | 0.40 | 72 | QH450 |
| 25 | 1 | 1 | 0.04 | 72 | QH451 |
| 26 | 0.1 | 0.1 | 0.04 | 24 | QH413 |
| 27 | 1 | 1 | 0.40 | 72 | QH416 |
| 28 | 1 | 10 | 0.40 | 72 | QH452 |
| 29 | 10 | 0 | 0 | 0 | -- |

**Supplementary Table 4. Impact of formulation and sex on differences in immune responses in the dose optimization DOE.**

| Response | Interaction (% variation in response due to interaction of sex and composition factors) | Sex (% variation in response due to sex) | Composition (% variation in response due to composition) | Interaction (p value) | Sex (p value) | Composition (p value) | 95% CI of difference male to female | Groups with significant difference (p<0.05) |
| --- | --- | --- | --- | --- | --- | --- | --- | --- |
| IFN-γ | 4.09 | 0.01 | 78.67 | 0.4363 | 0.7574 | **<0.0001** | -0.1566 to 0.1142 | none |
| IL-17A | 4.82 | 0.24 | 54.59 | 0.9855 | 0.4109 | **<0.0001** | -0.0613 to 0.1488 | none |
| stool IgA | 5.29 | 2.47 | 73.26 | 0.2933 | **0.0002 (female higher)** | **<0.0001** | -0.3120 to ‑0.1011 | Group 25 (female higher) |
| IgA ASC | 12.63 | 2.34 | 56.33 | **0.0140** | **0.0026 (female higher)** | **<0.0001** | -0.3371 to ‑0.0732 | none |
| IgG ASC | 15.86 | 0.88 | 54.96 | **0.0010** | 0.0605 | **<0.0001** | -0.2148 to 0.0047 | Group 11 (male higher) |
| serum IgG2a | 2.46 | 1.28 | 85.07 | 0.5803 | **0.0004 (female higher)** | **<0.0001** | -0.5216 to ‑0.1557 | none |
| serum IgG1 | 4.4 | 0.99 | 76.78 | 0.4409 | **0.0123 (female higher)** | **<0.0001** | -0.5016 to -0.0623 | none |
| serum IgGT | 2.5 | 1.45 | 83.67 | 0.6822 | **0.0003 (female higher)** | **<0.0001** | -0.5299 to ‑0.1612 | none |

Notes: Two-way ANOVA analysis was performed using the log-transformed values for each readout; bold text indicates p values <0.05.

**Supplementary Table 5. DOE model equation coefficients and model fit statistics.**

| Factor | Sqrt IFN-γ (Reduced Linear) | Log10 IL-17A (Linear) | Log10 Fecal IgA (Quadratic) | Log10 Bone Marrow ASC IgA (Reduced Quadratic) | Log10 Bone Marrow ASC IgG (Reduced Quadratic) | Log10 Serum IgGT (Reduced Quadratic) | Serum IgG2a/ IgG1 Log10 Ratio (Reduced Quadratic) |
| --- | --- | --- | --- | --- | --- | --- | --- |
| Intercept | 19.6851 | 1.7756 | 1.5316 | 0.8805 | 1.4348 | 4.3041 | 1.0113 |
| LecA | ***8.1771*** | ***0.2398*** | ***0.4742*** | ***0.3922*** | ***0.3558*** | ***1.1309*** | **-0.0407** |
| GLA |  | 0.0487 | 0.0415 | ***0.1473*** | **0.0842** | 0.1859 | -0.0270 |
| 3M-052 |  | 0.1094 | ***0.2471*** | ***0.2072*** | ***0.1411*** | ***0.7290*** | ***0.1437*** |
| Phospholipid |  | -0.0315 | 0.0265 | 0.0321 | **0.0836** |  | ***-0.0667*** |
| LecA*GLA |  |  | 0.1067 |  |  |  | 0.0364 |
| LecA*3M-052 |  |  | ***0.1801*** | ***0.2781*** | ***0.2239*** |  |  |
| LecA* Phospholipid |  |  | -0.0370 |  |  |  |  |
| GLA*3M-052 |  |  | -0.0730 |  |  | -0.2067 |  |
| GLA* Phospholipid |  |  | 0.0052 | 0.0954 |  |  | -0.0322 |
| 3M-052* Phospholipid |  |  | 0.0104 |  | **-0.0872** |  |  |
| LecA*LecA |  |  | ***-0.4311*** | **-0.2783** | **-0.1731** | ***-1.2474*** | ***0.1950*** |
| GLA*GLA |  |  | 0.0373 | 0.2289 |  | 0.4508 |  |
| 3M-052* 3M-052 |  |  | -0.2017 | **-0.2950** | **-0.1912** |  |  |
| Phospholipid* Phospholipid |  |  | 0.1836 |  |  |  | ***-0.1367*** |
| Model p-value | ***0.0014*** | **0.0101** | ***< 0.0001*** | ***< 0.0001*** | ***< 0.0001*** | ***< 0.0001*** | ***< 0.0001*** |
| Lack of Fit p-value | 0.0657 | 0.6059 | 0.5205 | 0.2817 | 0.4862 | 0.1821 | 0.3705 |
| Adjusted R^2^ | 0.3138 | 0.3367 | 0.8456 | 0.8328 | 0.8962 | 0.8894 | 0.8272 |
| Predicted R^2^ | 0.2277 | 0.1620 | 0.6539 | 0.6874 | 0.8262 | 0.8409 | 0.6979 |
| Adeq Precision | 6.2182 | 7.2282 | 10.0408 | 14.1351 | 16.6829 | 18.8378 | 13.6515 |

**p-value shading:** p>0.05, **p<0.05, *p<0.01*, *p<0.001*.**

**Supplementary Table 6. Confirmation of model validity using the proof-of-concept composition test point responses (experimental group #15).**

|  | **IFNγ (pg/ml)** | **IL-17A (pg/ml)** | **Stool IgA (Endpoint Titer)** | **Bone Marrow ASC IgA (#/10^6^ cells)** | **Bone Marrow ASC IgG (#/10^6^ cells)** | **Serum IgGT (Endpoint Titer)** | **Serum IgG2a/IgG1 Log_10_ Ratio** |
| --- | --- | --- | --- | --- | --- | --- | --- |
| Actual response | 709.0 | 143.6 | 146.4 | 8.0 | 32.2 | 99,990 | 1.04 |
| Predicted mean response (95% CI) | 739.0 (23.8 – 2110.6) | 138.6 (28.1 – 456.8) | 85.4 (22.4 - 254.8) | 26.5 (7.8 – 70.6) | 62.4 (28.9 – 121.3) | 287,335 (19,268 – 1,600,810) | 1.18 (1.01 – 1.34) |

**Supplementary Table 7. Experimental groups for the excipient composition immunogenicity study.**

| Group # | Description* | Phospholipid/ PEGylated Lipid Acyl Chain Length and Saturation** | Phospholipid: PEGylated Lipid Molar Ratio | Additional Excipients | Adjuvant Batch# |
| --- | --- | --- | --- | --- | --- |
| 1 | Antigen alone control | -- | -- | -- | -- |
| 2 | Same test composition as Experimental Group 15 in the dose optimization DOE | 16:0/18:0 (i.e.DPPC:DSPE-PEG) | 12.2 | -- | QH392 |
| 3 | Optimal composition predicted by the dose optimization DOE model | 16:0/18:0 | 12.2 | -- | QH527 |
| 4 | Suboptimal composition predicted by the dose optimization DOE model (0.1 µg 3M-052 dose) | 16:0/18:0 | 12.2 | -- | QH542 |
| 5 | Optimal composition plus tonicity agent | 16:0/18:0 | 12.2 | Glycerol (1.15 w/v%) | QH524 |
| 6 | Optimal composition plus viscosity agent | 16:0/18:0 | 12.2 | Microcrystalline cellulose/Carboxy-methylcellulose sodium (0.9 w/v%) | QH526 |
| 7 | Optimal composition plus antioxidant | 16:0/18:0 | 12.2 | α-tocopherol (0.005 w/v%) | QH525 |
| 8 | -- | 16:0/16:0 | 4.1 | -- | QH532 |
| 9 | -- | 18:1/18:1 | 12.2 | -- | QH540 |
| 10 | -- | 18:1/18:1 | 4.1 | -- | QH523 |
| 11 | -- | 14:0/14:0 | 4.1 | -- | QH537 |
| 12 | -- | 14:0/14:0 | 12.2 | -- | QH522 |
| 13 | -- | 16:0/16:0 | 36.6 | -- | QH530 |
| 14 | -- | 18:0/18:0 | 4.1 | -- | QH529 |
| 15 | -- | 16:0/16:0 | 12.2 | -- | QH531 |
| 16 | -- | 18:0/18:0 | 36.6 | -- | QH521 |
| 17 | -- | 18:1/18:1 | 36.6 | -- | QH539 |
| 18 | -- | 14:0/14:0 | 36.6 | -- | QH536 |
| 19 | -- | 18:0/18:0 | 12.2 | -- | QH528 |
| 20 | Suboptimal composition predicted by the dose optimization DOE model (0.5 µg LecA dose) | 16:0/18:0 | 12.2 | -- | QH531 |

*Unless otherwise noted, all groups received 10 µg LecA, 10 µg GLA, 4 µg 3M-052, 10 µg cholesterol, and 46 nmol phospholipid (31-36 µg depending on phospholipid). **14:0 refers to DMPC or DMPE-PEG, 16:0 refers to DPPC or DPPE-PEG, 18:0 refers to DSPC or DSPE-PEG, 18:1 refers to DOPC or DOPE-PEG.

**Supplementary Table 8. Physicochemical stability (actual values) of adjuvant formulations employed in the excipient composition immunogenicity experiment.**

| Group # | Description | % phospholipid loss after 6 months at 25C** | % PEGylated lipid loss after 6 months at 25C** | % cholesterol loss after 6 months at 25C | % 3M-052 loss after 4 months at 40C | % GLA loss after 3 months at 40C | | Drop in pH after 4 months at 40C | % Particle diameter increase after 6 months at 25C | Size polydispersity index increase after 6 months at 25C |
| --- | --- | --- | --- | --- | --- | --- | --- | --- | --- | --- |
| 1 | LecA alone | -- | -- | -- | -- | -- | -- | | -- | -- |
| 2 | POC | 34% | 64% | 99% | 14% | 64% | 2 | | 22% | 0 |
| 3 | PO | 26% | 44% | 100% | 0% | 12% | 0.59 | | 18% | 0 |
| 4 | PSO (less 3M-052) | 45% | 72% | 100% | 47% | 63% | 1.1 | | 10% | 0 |
| 5 | PO + tonicity agent | 27% | 41% | 100% | 0% | 10% | 0.42 | | 28% | 0 |
| 6 | PO + viscosity agent* | -- | -- | -- | -- | -- | -- | | -- | -- |
| 7 | PO + antioxidant | 15% | 20% | 0% | 1% | 14% | 0 | | 20% | 0.026 |
| 8 | DPPC, Low ratio | 17% | 50% | 100% | 12% | 33% | 2.14 | | 10% | 0.029 |
| 9 | DOPC, Mid ratio | 9% | 3% | 0% | 41% | 9% | 1.77 | | 2% | 0 |
| 10 | DOPC, Low ratio | 100% | 100% | 100% | 34% | 38% | 1.83 | | 35% | 0.206 |
| 11 | DMPC, Low ratio | 33% | 30% | 100% | 11% | 19% | 1.49 | | 28% | 0 |
| 12 | DMPC, Mid ratio | 27% | 41% | 100% | 4% | 19% | 0.48 | | 37% | 0 |
| 13 | DPPC, High ratio | 22% | 39% | 100% | 15% | 12% | 0.41 | | 100% | 0.583 |
| 14 | DSPC, Low ratio | 42% | 59% | 95% | 11% | 19% | 2.34 | | 16% | 0.082 |
| 15 | DPPC, Mid ratio | 23% | 38% | 100% | 13% | 10% | 0.6 | | 6% | 0 |
| 16 | DSPC, High ratio | 10% | 27% | 99% | 8% | 19% | 0.01 | | 73% | 0.255 |
| 17 | DOPC, High ratio | 6% | 7% | 0% | 37% | 1% | 1.67 | | 0% | 0 |
| 18 | DMPC, High ratio | 20% | 100% | 100% | 10% | 4% | 0.32 | | 100% | 0.657 |
| 19 | DSPC, Mid ratio | 25% | 94% | 100% | 0% | 8% | 0.78 | | 34% | 0 |
| 20 | PSO (less LecA) | 23% | 38% | 100% | 13% | 10% | 0.6 | | 6% | 0 |

In general, any negative values were represented as zero values in the table. PO: Predicted Optimal formulation from dose optimization DOE; PSO: Predicted Sub-Optimal formulations from dose optimization DOE; POC: Proof-of-Concept formulation (same as group #15 from the dose optimization DOE); DMPC, DPPC, DSPC, DOPC refers to acyl chain structure of liposomal lipids; High, Mid, Low refers to phospholipid:PEGylated lipid ratios. See Supplementary Table 5 for additional formulation composition details. *****The viscosity agent interfered with HPLC and particle size assays, preventing accurate data acquisition. **The 25C data at 6 months were calculated with respect to the 5C data at 6 months since time zero data was not available for many of the batches.

**Supplementary Table 9. Physicochemical stability desirability scores of adjuvant formulations employed in the excipient composition immunogenicity experiment.**

| Group # | Description | phospholipid stability (1)** | PEGylated lipid stability (1) | cholesterol stability (1) | 3M-052 stability (5) | GLA stability (4) | pH stability (3) | Particle diameter stability (5) | Size polydispersity index stability (2) | Overall Adjuvant Stability Desirability Score |
| --- | --- | --- | --- | --- | --- | --- | --- | --- | --- | --- |
| 7 | PO + antioxidant | 0.896 | 0.819 | 0.990 | 0.971 | 0.789 | 0.990 | 0.798 | 0.951 | 0.886 |
| 3 | PO | 0.787 | 0.575 | 0.010 | 0.982 | 0.825 | 0.743 | 0.810 | 0.990 | 0.687 |
| 5 | PO + tonicity agent | 0.778 | 0.605 | 0.010 | 0.990 | 0.855 | 0.814 | 0.713 | 0.990 | 0.683 |
| 15 | DPPC, Mid ratio | 0.813 | 0.640 | 0.010 | 0.717 | 0.852 | 0.739 | 0.936 | 0.990 | 0.669 |
| 20 | PSO (less LecA)*** | 0.813 | 0.640 | 0.010 | 0.717 | 0.852 | 0.739 | 0.936 | 0.990 | 0.669 |
| 12 | DMPC, Mid ratio | 0.772 | 0.605 | 0.010 | 0.917 | 0.712 | 0.789 | 0.626 | 0.990 | 0.628 |
| 19 | DSPC, Mid ratio | 0.795 | 0.075 | 0.010 | 0.990 | 0.887 | 0.663 | 0.660 | 0.990 | 0.598 |
| 17 | DOPC, High ratio | 0.990 | 0.952 | 0.990 | 0.216 | 0.990 | 0.291 | 0.990 | 0.990 | 0.591 |
| 11 | DMPC, Low ratio | 0.709 | 0.721 | 0.010 | 0.757 | 0.718 | 0.366 | 0.721 | 0.990 | 0.562 |
| 16 | DSPC, High ratio | 0.948 | 0.747 | 0.022 | 0.814 | 0.714 | 0.986 | 0.271 | 0.610 | 0.526 |
| 9 | DOPC, Mid ratio | 0.964 | 0.990 | 0.990 | 0.132 | 0.876 | 0.249 | 0.974 | 0.990 | 0.504 |
| 8 | DPPC, Low ratio | 0.873 | 0.515 | 0.010 | 0.732 | 0.497 | 0.094 | 0.889 | 0.947 | 0.450 |
| 14 | DSPC, Low ratio | 0.614 | 0.426 | 0.058 | 0.770 | 0.706 | 0.010 | 0.837 | 0.868 | 0.369 |
| 10 | DOPC, Low ratio | 0.010 | 0.010 | 0.010 | 0.279 | 0.410 | 0.223 | 0.643 | 0.683 | 0.241 |
| 2 | POC | 0.699 | 0.375 | 0.025 | 0.698 | 0.010 | 0.152 | 0.776 | 0.990 | 0.231 |
| 13 | DPPC, High ratio | 0.823 | 0.625 | 0.010 | 0.677 | 0.822 | 0.818 | 0.010 | 0.120 | 0.195 |
| 18 | DMPC, High ratio | 0.842 | 0.010 | 0.010 | 0.774 | 0.945 | 0.856 | 0.010 | 0.010 | 0.137 |
| 4 | PSO (less 3M-052) | 0.585 | 0.294 | 0.010 | 0.010 | 0.015 | 0.529 | 0.896 | 0.990 | 0.108 |
| 6 | PO + viscosity agent* | -- | -- | -- | -- | -- | -- | -- | -- | -- |
| 1 | LecA alone | -- | -- | -- | -- | -- | -- | -- | -- | -- |

PO: Predicted Optimal formulation from dose optimization DOE; PSO: Predicted Sub-Optimal formulations from dose optimization DOE; POC: Proof-of-Concept formulation (same as group #15 from the dose optimization DOE); DMPC, DPPC, DSPC, DOPC refers to acyl chain structure of liposomal lipids; High, Mid, Low refers to phospholipid:PEGylated lipid ratios. See Supplementary Table 7 for additional formulation composition details. *****The viscosity agent interfered with HPLC and particle size assays, preventing accurate data acquisition. **Numbers in parentheses in column titles refer to score weight in the overall desirability calculation. ***Same adjuvant formulation as experimental group #15. Color scale: higher desirability index values are light blue, lower desirability index values are dark blue.

**Supplementary Table 10. Impact of formulation and sex on differences in immune responses in the excipient composition immunogenicity experiment.**

| Response | Interaction (% variation in response due to interaction of sex and composition factors) | Sex (% variation in response due to sex) | Composition (% variation in response due to composition) | Interaction (p value) | Sex (p value) | Composition (p value) | 95% CI of difference male to female | Groups with significant difference (p<0.05) |
| --- | --- | --- | --- | --- | --- | --- | --- | --- |
| IFN-γ | 2.67 | 0.83 | 88.72 | 0.1300 | **0.0045 (female higher)** | **<0.0001** | -0.1603 to ‑0.0305 | Group 4 (female higher) |
| IL-17A | 10.92 | 1.99 | 54.31 | 0.1492 | **0.0304 (male higher)** | **<0.0001** | 0.0148 to 0.2909 | none |
| stool IgA | 6.94 | 0.49 | 71.41 | 0.1603 | 0.1758 | **<0.0001** | -0.2006 to 0.0373 | Group 19 (female higher) |
| IgA ASC | 10.52 | 1.33 | 54.74 | 0.1915 | 0.0784 | **<0.0001** | -0.0207 to 0.3772 | Group 8  (male higher) |
| IgG ASC | 8.60 | 1.65 | 53.82 | 0.4620 | 0.0588 | **<0.0001** | -0.0068 to 0.3654 | none |
| serum IgG2a | 2.07 | 0.42 | 87.74 | 0.5916 | 0.0662 | **<0.0001** | -0.2854 to 0.0094 | none |
| serum IgG1 | 2.37 | 0.02 | 84.14 | 0.7676 | 0.7304 | **<0.0001** | -0.1782 to 0.1254 | none |
| serum IgGT | 1.35 | 0.30 | 88.96 | 0.8924 | 0.1166 | **<0.0001** | -0.2581 to 0.0291 | none |

Notes: Two-way ANOVA analysis was performed using the log-transformed values for each readout; bold text indicates p values <0.05.

**Supplementary Table 11. Impact of time point and sex on differences in immune responses in the immunogenicity durability experiment.**

| Response (Group) | Statistical Model | Interaction (p value) | Sex (p value) | Time or Composition (p value) |
| --- | --- | --- | --- | --- |
| IFN-γ (all groups) | Two-Way ANOVA | 0.7821 | 0.1278 | 0.0629 (Composition) |
| stool IgA (LecA) | Two-Way ANOVA | 0.7271 | 0.7087 | 0.2535 (Time) |
| stool IgA (LecA + PS) | Two-Way ANOVA | 0.5012 | 0.7543 | 0.2260  (Time) |
| stool IgA (LecA + PP) | Mixed Effects | 0.3122 | **0.0407 (female higher)** | 0.0908  (Time) |
| stool IgA (LecA + SS) | Mixed Effects | 0.1275 | **0.0170 (female higher)** | **0.0257**  **(Time)** |
| serum IgG1 (LecA) | Two-Way ANOVA | -- | -- | -- |
| serum IgG1 (LecA + PS) | Two-Way ANOVA | **0.0475** | 0.5633 | **0.0342**  **(Time)** |
| serum IgG1 (LecA + PP) | Mixed Effects | 0.1441 | 0.5006 | **<0.0001**  **(Time)** |
| serum IgG1 (LecA + SS) | Mixed Effects | 0.5369 | 0.0779 | **<0.0001**  **(Time)** |
| serum IgG2a (LecA) | Two-Way ANOVA | -- | -- | -- |
| serum IgG2a (LecA + PS) | Two-Way ANOVA | 0.3365 | 0.4424 | **0.0016**  **(Time)** |
| serum IgG2a (LecA + PP) | Mixed Effects | **0.0419** | 0.691 | **0.0019**  **(Time)** |
| serum IgG2a (LecA + SS) | Mixed Effects | 0.6018 | **0.0007 (female higher)** | **0.0456**  **(Time)** |
| serum IgGT (LecA) | Two-Way ANOVA | 0.4155 | 0.3559 | 0.3559  (Time) |
| serum IgGT (LecA + PS) | Two-Way ANOVA | **0.0233** | 0.8241 | **0.0003**  **(Time)** |
| serum IgGT (LecA + PP) | Mixed Effects | 0.2030 | 0.4075 | **<0.0001**  **(Time)** |
| serum IgGT (LecA + SS) | Mixed Effects | 0.3171 | **0.0002 (female higher)** | **0.0003**  **(Time)** |

Notes: Values were log-transformed prior to analysis. Two-way ANOVA analysis was performed unless missing values were present in which case mixed-effects analysis was performed. The second factor was time for all readouts except IFNγ, where composition was the second factor since there was not more than one time point for that readout. Serum IgG1 and serum IgG2a responses were at the lower limit for all mice immunized with LecA alone. Bold text indicates p values <0.05.

**Supplementary Table 12. Single TLR ligand formulations (compare to ‘PP’ row in Table 5).**

| **GLA (mg/ml)** | **3M-052 (mg/ml)** | **Primary phospho-lipid (mg/ml)** | **PEG-ylated phospho-lipid (mg/ml)** | **Choles-terol (mg/ ml)** | **α-toco-pherol (mg/ ml)** | **Buffer (ammo-nium phos-phate)** | **pH** | **Particle diameter (Z-ave, nm)** | **Size Polydisp-ersity Index (PdI)** |
| --- | --- | --- | --- | --- | --- | --- | --- | --- | --- |
| -- | 0.32 +/- 0.00 | 3.32 +/- 0.06 (DPPC) | 1.00 +/- 0.02 (DPPE-PEG2000) | 1.01 +/- 0.02 | 0.06 +/- 0.00 | 25 mM | 5.83 | 87.1 +/- 3.4 | 0.559 +/- 0.012 |
| 0.96 +/- 0.01 | -- | 3.32 +/- 0.03 (DPPC) | 0.96 +/- 0.01 (DPPE-PEG2000) | 0.95 +/- 0.01 | 0.06 +/- 0.00 | 25 mM | 5.78 | 68.9 +/- 0.8 | 0.186 +/- 0.018 |


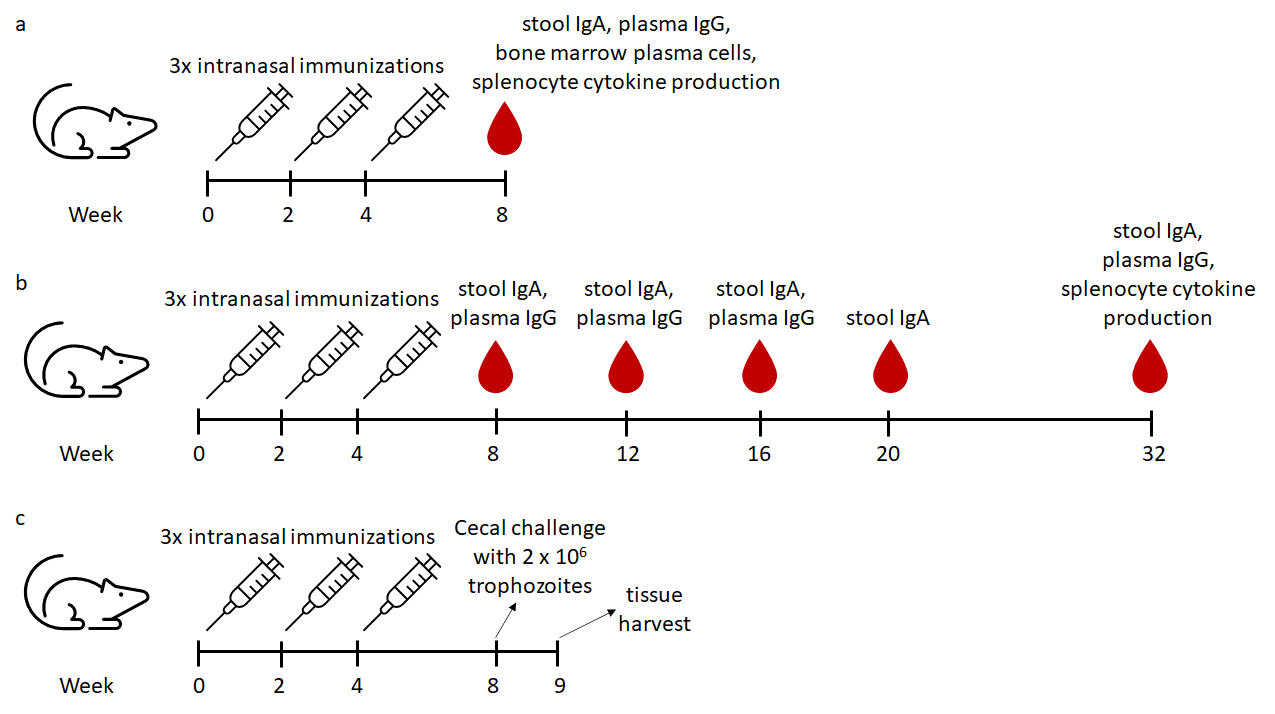


**Supplementary Figure 1. Mouse immunization and immunological readout schedules. (a) Dose optimization DOE and excipient composition immunogenicity studies. (b) Immune response durability study. (c) Protective efficacy studies.**


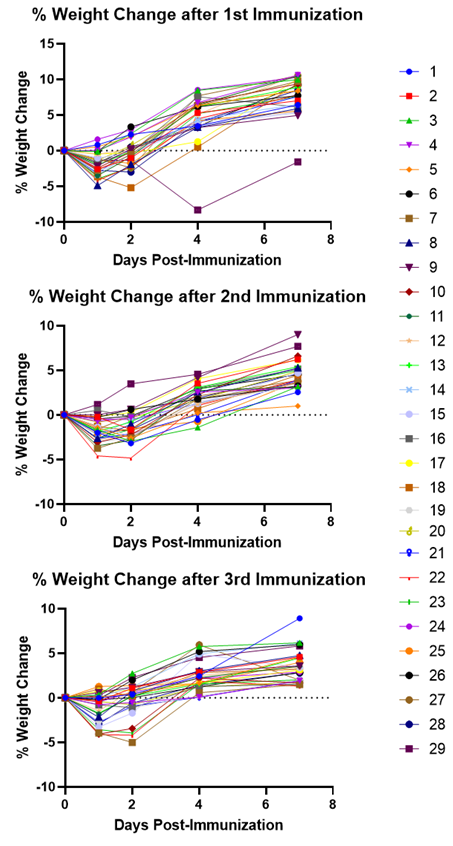


**Supplementary Figure 2. Mouse weight change following each immunization in the dose optimization DOE. Mean values are represented. Each experimental group consisted of 6 mice except for group 29 where one animal was sacrificed due to weight loss 6 days after the first immunization, and in group 15 two mice were omitted from the top panel as they were not measured for Day 0 weight.**

**a**


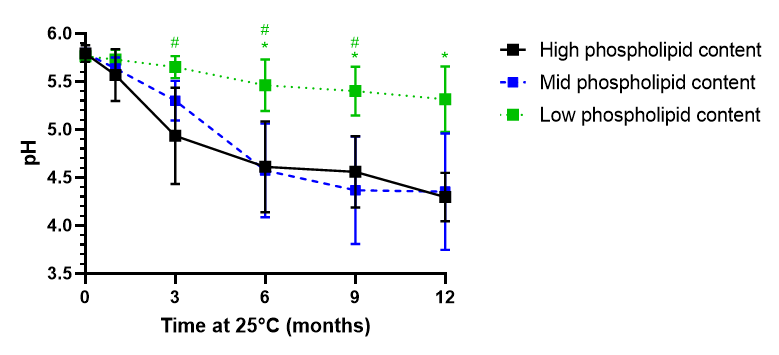


**b**


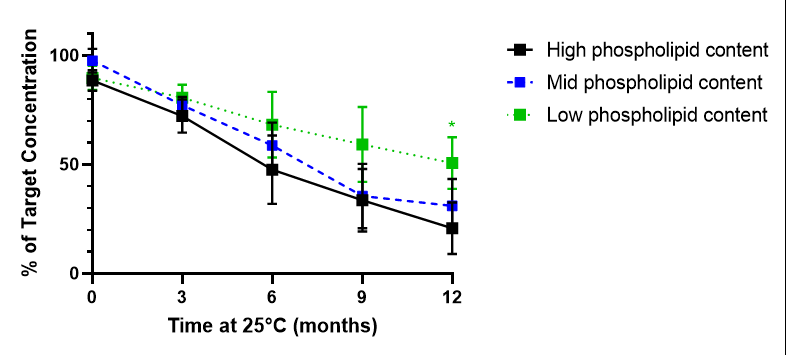


**Supplementary Figure 3. Higher phospholipid concentrations result in decreased stability. (a) Effect of initial phospholipid concentrations on formulation pH stability with formulations stored at 25°C. (b) Effect of initial phospholipid concentrations on DPPC stability with formulations stored at 25°C. For both panels, high, mid, and low phospholipid concentrations correspond to the target values of 21.6, 7.2, and 2.4 mg/ml DPPC, respectively. Symbols represent the mean +/- s.d of 3-6 adjuvant batches at each time point. *p<0.05 vs. high phospholipid content by two-way ANOVA; #p<0.05 vs. mid phospholipid content by two-way ANOVA.**


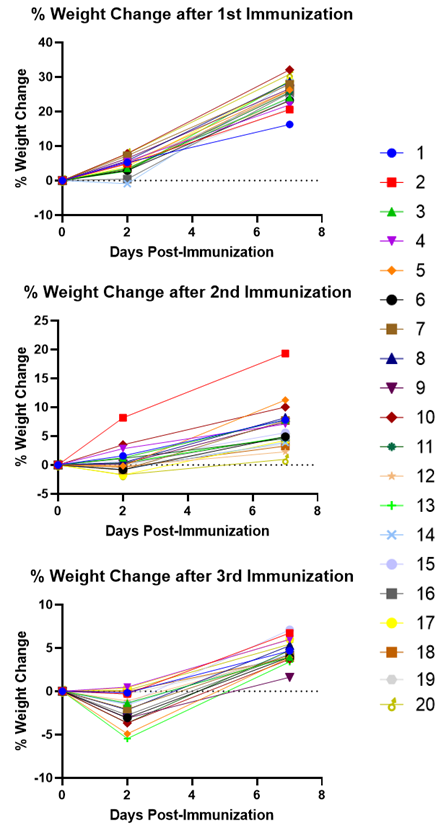


**Supplementary Figure 4. Mouse weight change following each immunization in the excipient optimization immunogenicity experiment. Mean values are represented. Each experimental group consisted of 6 mice.**

**a**


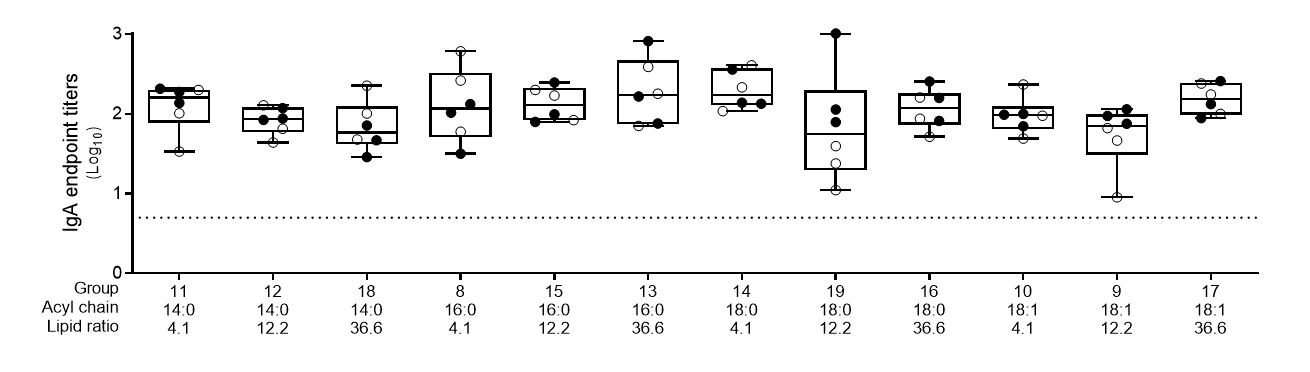


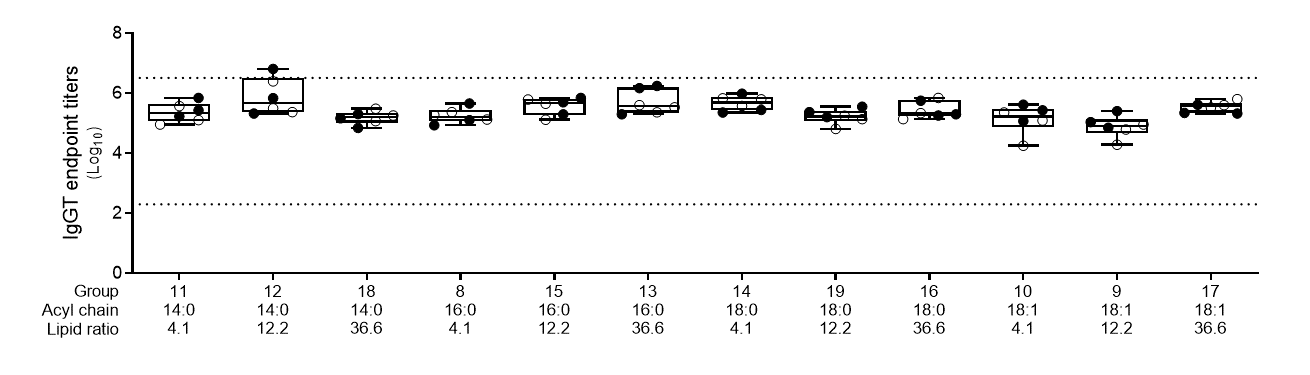


**b**


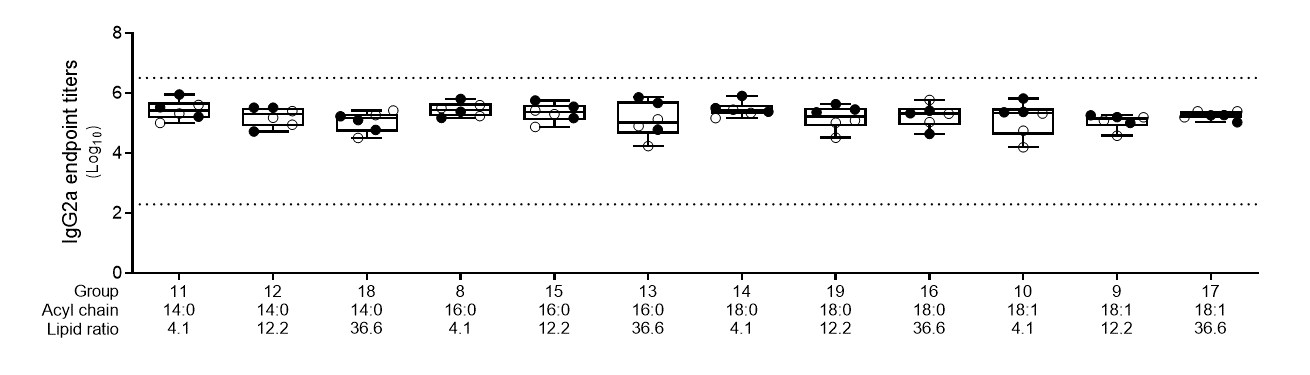


**c**


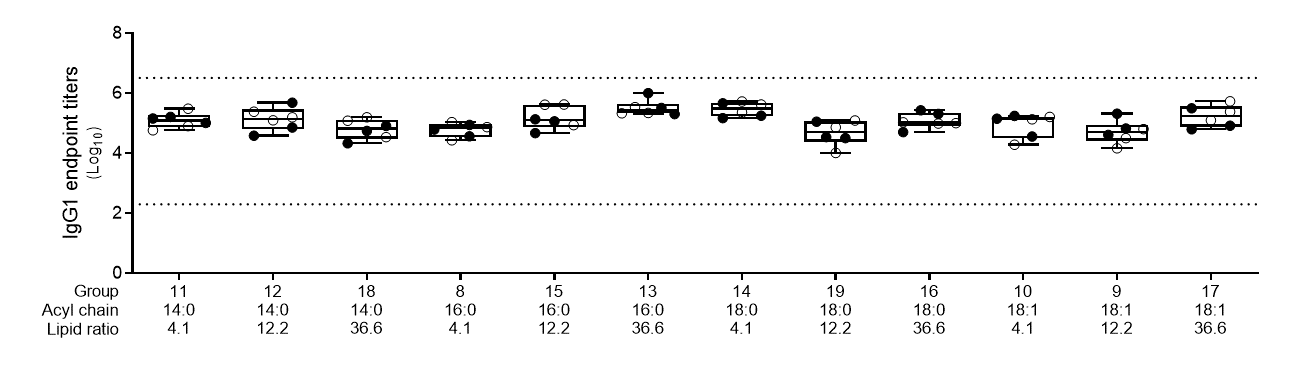


**d**


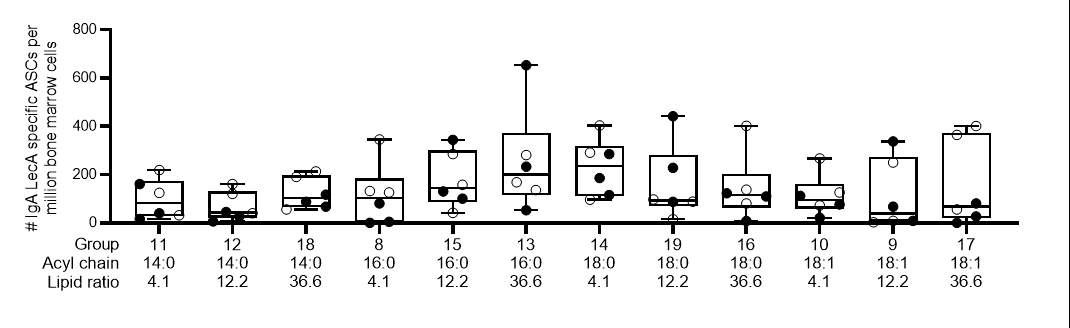


**e**


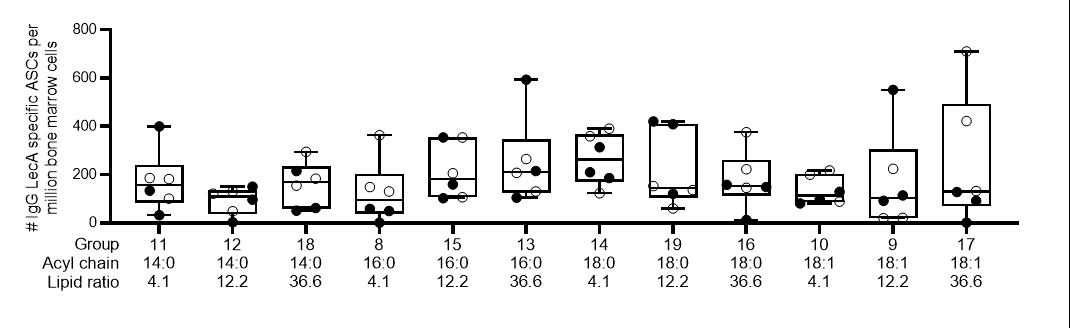


**f**


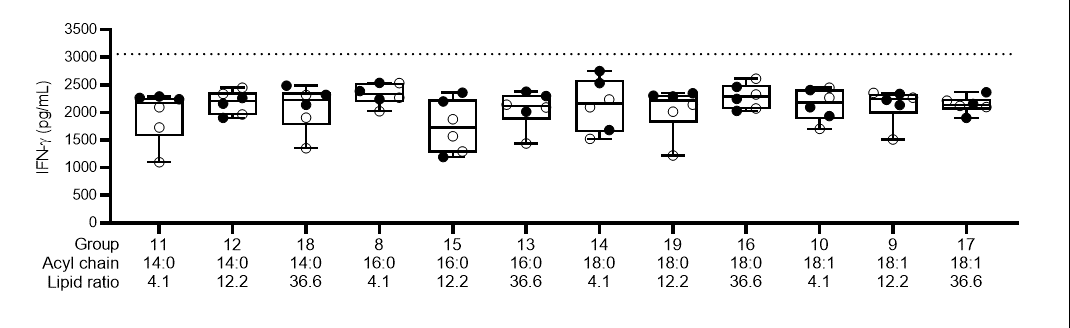


**g**


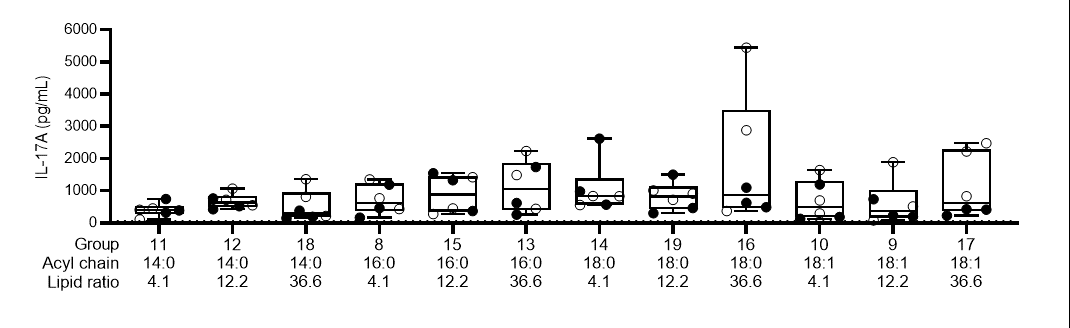


**h**

**Supplementary Figure 5. Antibody and cellular immune responses elicited in mice 4 weeks after the last immunization in groups 8-19 of the excipient composition immunogenicity study (see Supplementary Table 3). (a) stool LecA IgA titers, (b) plasma LecA IgG total titers, (c) plasma LecA IgG2a titers, (d) plasma LecA IgG1 titers, (e) LecA-specific IgA-secreting bone marrow cells, (f) LecA-specific IgG-secreting bone marrow cells, (g) LecA-specific IFNγ secretion by splenocytes, and (h) LecA-specific IL-17A secretion by splenocytes. For all panels, females are represented by closed circles and males by open circles. The box-whisker plots represent the median values (bars), the 25^th^-75^th^ percentiles (boxes), and the minimum and maximum values (whiskers), with all points shown. Dotted lines indicate the upper and lower limits of quantitation; responses outside of these ranges were arbitrarily set to 2x the upper limit or 0.5x the lower limit, respectively.**
